# One-Cell Metabolic Phenotyping and Sequencing of Soil Microbiome by Raman-Activated Gravity-Driven Encapsulation (RAGE)

**DOI:** 10.1128/mSystems.00181-21

**Published:** 2021-05-27

**Authors:** Xiaoyan Jing, Yanhai Gong, Teng Xu, Yu Meng, Xiao Han, Xiaolu Su, Jianmei Wang, Yuetong Ji, Yuandong Li, Zhongjun Jia, Bo Ma, Jian Xu

**Affiliations:** a Single-Cell Center, CAS Key Laboratory of Biofuels, Shandong Key Laboratory of Energy Genetics and Shandong Institute of Energy Research, Qingdao Institute of BioEnergy and Bioprocess Technology, Chinese Academy of Sciences, Qingdao, Shandong, China; b State Key Laboratory of Soil and Sustainable Agriculture, Institute of Soil Science, Chinese Academy of Sciences, Nanjing, Jiangsu, China; c Laboratory for Marine Biology and Biotechnology, Qingdao National Laboratory for Marine Science and Technology, Qingdao, Shandong, China; d Qingdao Single-cell Biotechnology Co., Ltd., Qingdao, Shandong, China; e University of Chinese Academy of Sciences, Beijing, China; University of California, Davis

**Keywords:** soil microbiome, Raman-activated gravity-driven single-cell encapsulation and sequencing (RAGE-Seq), single-cell genomics, D_2_O, carotenoids

## Abstract

Soil harbors arguably the most metabolically and genetically heterogeneous microbiomes on Earth, yet establishing the link between metabolic functions and genome at the precisely one-cell level has been difficult. Here, for mock microbial communities and then for soil microbiota, we established a Raman-activated gravity-driven single-cell encapsulation and sequencing (RAGE-Seq) platform, which identifies, sorts, and sequences precisely one bacterial cell via its anabolic (incorporating D from heavy water) and physiological (carotenoid-containing) functions. We showed that (i) metabolically active cells from numerically rare soil taxa, such as *Corynebacterium* spp., *Clostridium* spp., *Moraxella* spp., *Pantoea* spp., and Pseudomonas spp., can be readily identified and sorted based on D_2_O uptake, and their one-cell genome coverage can reach ∼93% to allow high-quality genome-wide metabolic reconstruction; (ii) similarly, carotenoid-containing cells such as *Pantoea* spp., *Legionella* spp., *Massilia* spp., Pseudomonas spp., and *Pedobacter* spp. were identified and one-cell genomes were generated for tracing the carotenoid-synthetic pathways; and (iii) carotenoid-producing cells can be either metabolically active or inert, suggesting culture-based approaches can miss many such cells. As a Raman-activated cell sorter (RACS) family member that can establish a metabolism-genome link at exactly one-cell resolution from soil, RAGE-Seq can help to precisely pinpoint “who is doing what” in complex ecosystems.

**IMPORTANCE** Soil is home to an enormous and complex microbiome that features arguably the highest genomic diversity and metabolic heterogeneity of cells on Earth. Their *in situ* metabolic activities drive many natural processes of pivotal ecological significance or underlie industrial production of numerous valuable bioactivities. However, pinpointing “who is doing what” in a soil microbiome, which consists of mainly yet-to-be-cultured species, has remained a major challenge. Here, for soil microbiota, we established a Raman-activated gravity-driven single-cell encapsulation and sequencing (RAGE-Seq) method, which identifies, sorts, and sequences at the resolution of precisely one microbial cell via its catabolic and anabolic functions. As a Raman-activated cell sorter (RACS) family member that can establish a metabolism-genome link at one-cell resolution from soil, RAGE-Seq can help to precisely pinpoint “who is doing what” in complex ecosystems.

## INTRODUCTION

Soil is home to an enormous and complex microbiome, which features arguably the highest genomic diversity and metabolic heterogeneity of cells on Earth (1). The *in situ* metabolic activities of the soil, individually or collaboratively mediated by its prokaryotic and eukaryotic residents, drive many natural processes of pivotal ecological significance or underlie industrial production of numerous compounds of value (2–4). However, pinpointing “who is doing what” in a soil microbiome, which consists of mainly yet-to-be-cultured species, has remained a major challenge, which is confounded by multiple factors. (i) Every microbial cell’s metabolic phenotype can be distinct, yet metagenome/transcriptome/metabolome technologies are usually unable to precisely assign a metabolic activity to individual cells. (ii) Each cell’s genotype is also theoretically different, even for microbial residents that live in physical proximity (in fact, the majority of numerically rare species in the soil microbiome live in floating lifestyles as individual cells rather than aggregated biofilms of clustering population) (5–7). (iii) A metabolic phenotype or activity can be underpinned by multiple genomes and vice versa (8). (iv) Even for those cells that can be cultured, their activity in the test tube can be different (or even irrelevant) from their *in situ* role (9). Therefore, establishing the link between metabolic phenotypes and individual genomes (i.e., “who” is doing “what”) at the resolution of one cell is of utmost importance to mechanistic dissection and resource mining of the soil microbiome (10).

Raman-activated cell sorting and sequencing (RACS-Seq) can potentially address this problem (11–14). In RACS-Seq, single-cell Raman spectra (SCRS), an intrinsic biochemical fingerprint of individual cells, are used as a proxy of metabolic phenotype. When coupled with stable isotope probing (SIP-Raman), SCRS can reveal the degree of cellular substrate intake (e.g., ^13^C, ^15^N, and D) (10, 11, 15–19). For example, via heavy water (D_2_O) feeding, a treatment that does not change the substrate pool, the general metabolic activity of cells can be tracked via the Raman shift at the C-D (carbon-deuterium vibration) band in 2,040 to 2,300 cm^−1^ (2, 16–18, 20, 21). On the other hand, SCRS can reveal cellular biosynthetic profiles, such as carotenoids (22, 23), starch (24), protein (24), triacylglycerols (24–26), and other Raman-sensitive compounds.

After SCRS acquisition, to establish the phenotype-genotype links, individual cells of target SCRS can then be sorted via RACS and sequenced (14, 27, 28). Various RACS techniques were introduced for this purpose, such as Raman-activated cell ejection (RACE) (29, 30). By plating cells on a solid basis, acquiring SCRS tandemly, and then ejecting them individually into receiving wells, RACE has been applied for analyzing soil (29), seawater (22, 23), and human intestinal samples (20). On the other hand, Raman tweezers which acquire SCRS and sort individual cells in an aquatic phase were tested in soil (21) and mouse fecal samples (18).

However, linking single-cell sequencing to RACS has not been trivial. One key challenge in microbiome RACS-Seq has been the inability to establish the link between metabolic phenotypes and individual genomes at precisely one-cell resolution, due to technological hurdles. First, the small size (usually <5 μm in diameter) and light weight (picogram level) of a bacterial cell can hinder precise tracking, reliable manipulation, and ready transfer of precisely one cell (31). For example, the inability to readily confirm the presence of an ejected cell greatly reduces the RACE-Seq success rate (29). Second, the bias in multiple displacement amplification (MDA) can compromise the completeness of *de novo* one-cell genome assemblies (32). For example, to identify carbon-fixing bacteria from surface seawater of the Yellow Sea, success rate and genome coverage of individual cells isolated via RACE were so low that >30 cells of target SCRS had to be pooled into an MDA reaction mixture (22). Moreover, in the Red Sea samples, genome coverages of one-bacterial-cell reactions ranged from 4.17% to 8.18%, while those of 3- to 8-cell reactions were all <20% (23). Although we recently showed that introducing oils prior to MDA can elevate the success rate of 2- to 5-cell RACE-Seq reactions (cells pooled prior to MDA) for soil microbiota (29), success for precisely one-cell RACS-Seq for such complex environmental microbiota has not been reported yet.

To tackle this challenge, here for soil microbiota we established a one-cell RACS-Seq workflow via Raman-activated gravity-driven single-cell encapsulation and sequencing (RAGE-Seq) (33), which couples aqueous-phase SCRS acquisition and sorting with microdroplet-based single-cell lysis, MDA, and then whole-genome sequencing (WGS). We showed that the workflow can identify, sort, and sequence at precisely one-cell resolution, based on the cell’s D_2_O-assimilating and carotenoid-producing activities. Specifically, genome coverage of ∼90% can be achieved from just one soil bacterial cell that was functionally profiled for metabolic activities via its SCRS. Such an ability to profile and correlate bacterial metabolic phenome and high-quality genome sequences at precisely one-cell resolution is pivotal to unambiguously establishing the phenotype-genotype links in soil microbiomes, which thus should accelerate mechanistic dissection and bioresource mining from the soil and other complex ecosystems.

## RESULTS

### Benchmarking one-cell RAGE-Seq via mock microbial communities.

RAGE-Seq addresses the challenges of small cell size and weight and biased MDA of microbial single-cell DNA by screening individual cells via acquiring SCRS in an aquatic, vitality-preserving environment and then precisely packaging the target cell (i.e., with characteristic SCRS) into a picoliter droplet that can then be readily exported in a precisely indexed, “one-cell-one-tube” manner (33). However, our past demonstration of RAGE-Seq was based only on Escherichia coli (both pure-cultured lab strains and individual cells directly from clinical urine samples) (33). To develop a precisely one-cell RAGE-Seq procedure for environmental microbiota, which are typically much more diverse and complex, we employed soil as a model. We are interested in sorting from soil (i) metabolically active cells, which can be distinguished based on a broad Raman band that appears in the SCRS between 2,040 and 2,300 cm^−1^ (peaked at ∼2,157 cm^−1^; i.e., the C-D stretching vibrations shifted from the C-H stretching vibrations at 2,800 to 3,200 cm^−1^ [18] after deuterium incorporation from D_2_O into biomass via NADPH-mediated H/D exchange reactions), and (ii) carotenoid-producing cells, which can be recognized by characteristic SCRS bands of carotenoids in 1,500 to 1,550, 1,150 to 1,170, and 1,000 to 1,020 cm^−1^ (i.e., in-phase C=C [*v*3], C–C stretching [*v*2] vibrations of the polyene chain and in-plane rocking mode of CH_3_ groups attached to the polyene chain [*v*1] [15, 22, 34, 35]).

**FIG 1:**
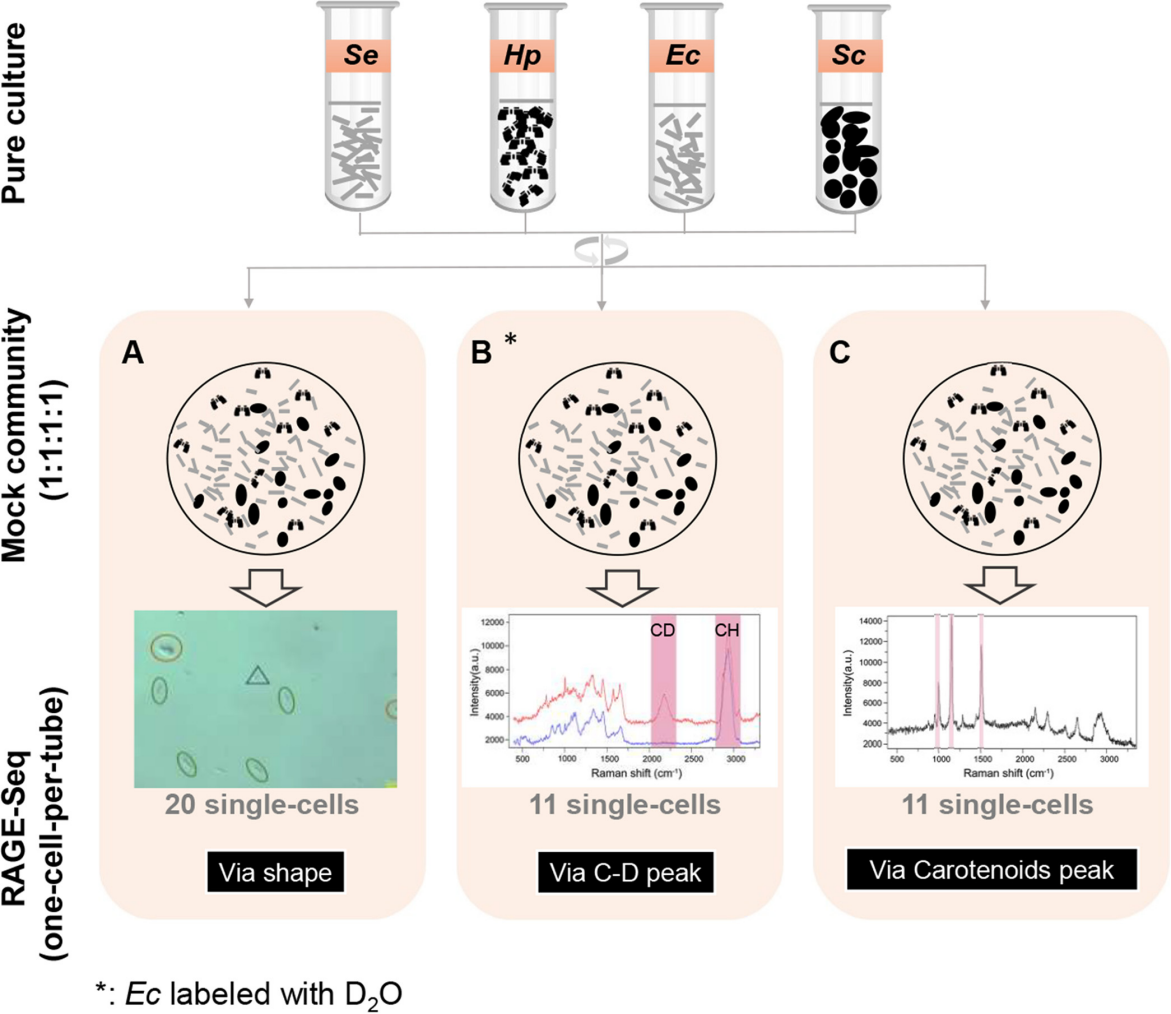
Benchmarking RAGE-Seq via a series of mock microbiota that include *S. elongatus* PCC7942 (*Se*), H. pylori ATCC 26695 (*Hp*), E. coli K-12 DH5α (*Ec*), and S. cerevisiae BY4742 (*Sc*). (A) Experiment A (for testing the specificity of RAGE-Seq via shape). Cells were sorted from the mock community based on different cellular morphology or full spectrum of SCRS and then sequenced. (B) Experiment B (for testing the specificity of sorting D_2_O-band-containg cells). Prior to the mixing, *Ec* was fed the D_2_O (to probe the metabolic activities of *Ec*). Cells that exhibited C-D peaks in SCRS were sorted and sequenced. (C) Experiment C (for testing the specificity of sorting carotenoid-band-containing cells). Cells were sorted from the mock community based on the presence of the carotenoid peaks and sequenced. In all three experiments above, the four types of cells were mixed in an equal ratio prior to performing SCRS acquisition and RAGE-Seq. All the RAGE-Seq reactions are one cell per tube.

**FIG 2:**
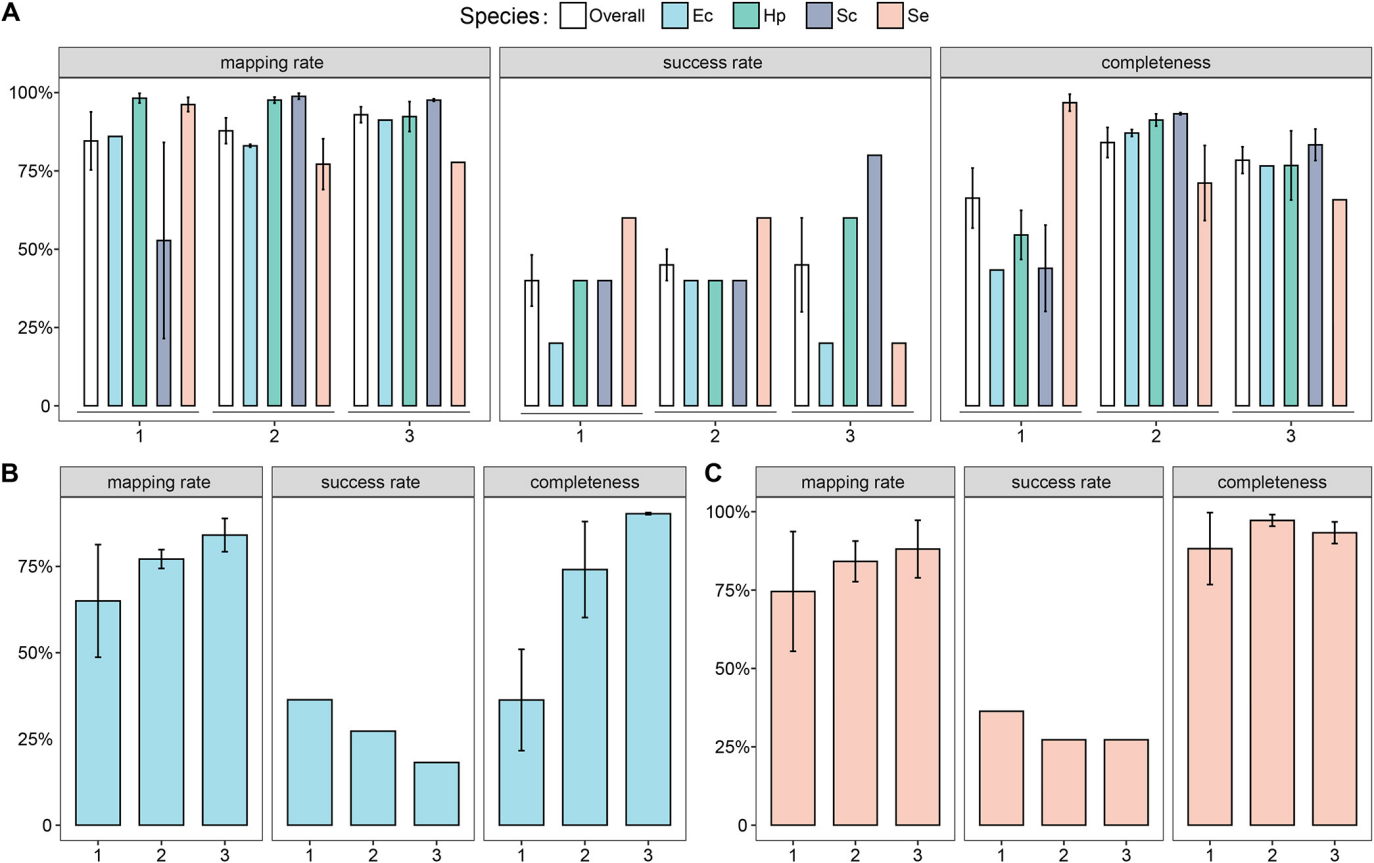
Validation of one-cell RAGE-Seq method performance via a synthetic four-species mock microbiota. (A) Results based on criterion A, which sorted via cell morphology. (B) Results based on criterion B, which sorted via the C-D peak of SCRS. (C) Results based on criterion C, which sorted via the carotenoid peak of SCRS. The mock microbiota consists of the prokaryote E. coli (Ec), the prokaryote H. pylori (Hp), the eukaryote S. cerevisiae (Sc), and the photosynthetic prokaryote *S. elongatus* (Se), mixed in equal abundance. For the three different sorting criteria, 20 cells, 11 cells, and 11 cells were sorted and sequenced per experiment, respectively, and three biological replicates were performed for each of the experiments. For each experiment, success rate (i.e., number of 16S sequencing-validated SAGs/total number of sorted cells), mapping rate, and genome completeness of the post-RAGE one-cell genome sequencing reactions were assessed (Materials and Methods).

To benchmark the performance of RAGE-Seq for such metabolic phenotypes from microbiota, we first constructed a series of mock microbial communities (see Fig. S1A and B in the supplemental material), consisting of Escherichia coli K-12 DH5α (*Ec*), Helicobacter pylori ATCC 26695 (*Hp*), Synechococcus elongatus PCC7942 (*Se*), and one fungus, Saccharomyces cerevisiae BY4742 (*Sc*), in a 1:1:1:1 ratio. Then, three series of experiments were designed to test the specificity of RAGE-Seq, via sorting the mock communities based on cell morphology (experiment A, Fig. 1A), the C-D peak of SCRS (experiment B, Fig. 1B), or the carotenoid peak of SCRS (experiment C, Fig. 1C), respectively. For each of the criteria, 20 cells, 11 cells, and 11 cells, respectively, per experiment (in triplicates) were sorted and sequenced using a RAGE chip (Fig. S1C; Materials and Methods). Thus, for the synthetic four-species mock microbiota, nine RAGE-Seq experiments in three biological replicates were performed, which sorted and sequenced 126 individual cells in total (Fig. 2A to C; Table 1; Materials and Methods).

**TABLE 1:** Benchmarking the performance of RAGE-Seq using a four-species mock microbiota^*a*^

Expt series	Sample ID	WGS reads mapped to reference genome (%)						One-cell WGS assembly				WGS consistent with sorting criteria	Success rate (%)
		*Hp*	*Se*	*Sc*	*Ec*	%Hit_no_genomes	Avg mapping rate for each species (%)	Taxonomy of bins based on sequence	Genome completeness (%)	Avg genome completeness for each species (%)	% contaminant bins		
A-1	A-1-Se1	0.13	**99.05**	0.00	0.08	0.78		*Se*	99.66		≤16.67	Yes	
	A-1-Se3	0.07	**91.68**	0.02	0.05	8.25	96.20	*Se*	91.38	96.78	≤4.17	Yes	60.00
	A-1-Se5	0.14	**97.86**	0.00	0.12	2.12		*Se*	99.29		≤4.17	Yes	
	A-1-Hp2	**99.76**	0.06	0.03	0.04	0.10	98.23	*Hp*	46.74	54.57	≤6.46	Yes	40.00
	A-1-Hp4	**96.69**	0.22	0.00	0.35	3.09		*Hp*	62.40		≤12.36	Yes	
	A-1-Sc3	0.01	0.09	**21.48**	0.03	78.34	52.79	*Sc*	57.70	43.92	≤9.95	Yes	40.00
	A-1-Sc4	0.60	0.84	**84.10**	0.59	14.59		*Sc*	30.14		≤8.43	Yes	
	A-1-Ec2	0.04	0.02	0.00	**86.01**	13.73	86.01	*Ec*	43.36	43.36	≤1.72	Yes	20.00
	A-1-NC	0.03	0.10	0.00	0.00	**99.53**					≤4.17	Yes	
A-2	A-2-Se2	0.21	**82.55**	0.08	0.13	16.81	77.16	*Se*	94.90	71.13	≤4.04	Yes	60.00
	A-2-Se3	12.83	**61.22**	0.01	0.05	25.85		*Se*	56.60		≤4.17	Yes	
	A-2-Se5	0.09	**87.70**	0.00	0.12	12.11		*Se*	61.90		≤4.17	Yes	
	A-2-Hp1	**98.58**	0.26	0.49	0.50	0.79	97.61	*Hp*	89.30	91.25	≤1.72	Yes	40.00
	A-2-Hp5	**96.64**	0.08	0.28	0.42	0.42		*Hp*	93.20		≤27.56	Yes	
	A-2-Sc1	0.00	0.00	**99.79**	0.00	0.18	98.83	*Sc*	92.90	93.25	≤0.00	Yes	40.00
	A-2-Sc3	0.01	0.00	**97.86**	0.00	2.06		*Sc*	93.60		≤5.42	Yes	
	A-2-Ec3	1.97	0.30	0.06	**82.54**	14.55	83.02	*Ec*	86.00	87.10	≤18.43	Yes	40.00
	A-2-Ec4	1.76	0.14	0.04	**83.50**	14.02		*Ec*	88.20		≤0.31	Yes	
A-3	A-3-Se1	0.01	**77.73**	0.18	0.04	21.96	77.73	*Se*	65.80	65.80	≤4.17	Yes	20.00
	A-3-Hp2	**96.39**	0.09	0.90	0.13	2.50	92.33	*Hp*	94.20	76.73	≤13.79	Yes	60.00
	A-3-Hp4	**82.81**	2.11	0.07	4.60	16.13		*Hp*	56.30		≤14.25	Yes	
	A-3-Hp5	**97.80**	0.75	0.22	0.83	1.93		*Hp*	79.70		≤0.16	Yes	
	A-3-Sc2	0.47	0.01	**97.10**	0.02	2.29	97.61	*Sc*	86.30	83.33	≤4.17	Yes	80.00
	A-3-Sc3	0.85	0.00	**98.63**	0.00	0.43		*Sc*	68.40		≤13.03	Yes	
	A-3-Sc4	0.05	0.00	**97.85**	0.00	2.05		*Sc*	89.60		≤7.02	Yes	
	A-3-Sc5	0.19	0.00	**96.85**	0.00	2.87		*Sc*	89.00		≤5.26	Yes	
	A-3-Ec5	0.32	0.26	0.06	**91.22**	8.09	91.22	*Ec*	76.60	76.60	≤7.44	Yes	20.00
	A-3-NC	0.00	0.02	0.06	0.26	**96.71**					≤2.90	Yes	
B-1	B-1-Ec2	0.29	0.28	0.00	**80.11**	19.23	65.00	*Ec*	80.09	36.30	≤3.45	Yes	36.36
	B-1-Ec4	1.33	0.73	0.08	**66.35**	33.55		*Ec*	25.91		≤10.34	Yes	
	B-1-Ec7	0.00	0.01	0.79	**19.13**	79.75		*Ec*	20.81		≤4.67	Yes	
	B-1-Ec10	0.20	0.14	0.00	**94.41**	1.29		*Ec*	18.37		≤0.96	Yes	
	B-1-NC	0.00	0.00	0.00	0.00	**99.95**					≤5.33	Yes	
B-2	B-2-Ec3	0.28	0.42	0.01	**82.25**	16.64	77.15	*Ec*	90.90	74.10	≤57.07	Yes	27.27
	B-2-Ec5	0.33	0.61	0.02	**72.95**	26.09		*Ec*	84.90		≤36.07	Yes	
	B-2-Ec8	0.19	0.46	0.02	**76.24**	22.05		*Ec*	46.50		≤30.83	Yes	
	B-2-NC	0.00	0.02	0.05	0.06	**99.41**					≤5.33	Yes	
B-3	B-3-Ec1	0.49	0.73	0.04	**79.26**	19.54	84.09	*Ec*	90.60	90.30	≤44.26	Yes	18.18
	B-3-Ec8	0.27	0.27	0.00	**88.91**	10.23		*Ec*	90.00		≤19.65	Yes	
C-1	C-1-Se1	0.03	**17.49**	0.14	0.17	82.12	74.58	*Se*	53.80	88.24	≤10.64	Yes	36.36
	C-1-Se5	0.15	**96.61**	0.07	0.18	3.09		*Se*	99.69		≤10.10	Yes	
	C-1-Se8	0.14	**95.04**	0.06	0.19	4.63		*Se*	99.73		≤9.64	Yes	
	C-1-Se10	0.15	**89.16**	0.00	0.12	10.69		*Se*	99.72		≤7.86	Yes	
	C-1-NC	0.00	0.00	0.00	0.00	**98.23**					≤0.31	Yes	
C-2	C-2-Se6	0.63	**80.63**	0.00	13.51	5.23	84.16	*Se*	99.70	97.23	≤12.73	Yes	27.27
	C-2-Se7	17.61	**75.14**	0.00	0.10	7.06		*Se*	93.60		≤16.67	Yes	
	C-2-Se10	0.15	**96.72**	0.00	1.29	2.08		*Se*	98.40		≤8.07	Yes	
	C-2-NC	0.00	0.00	1.15	0.01	**74.40**					≤14.66	Yes	
C-3	C-3-Se2	0.31	**98.27**	0.06	0.14	1.27	88.10	*Se*	96.40	93.30	≤8.33	Yes	27.27
	C-3-Se5	0.09	**96.24**	0.04	0.09	3.66		*Se*	97.10		≤4.17	Yes	
	C-3-Se9	0.08	**69.78**	0.05	0.01	29.95		*Se*	86.40		≤10.34	Yes	
	C-3-NC	0.00	0.00	0.78	0.24	**95.47**					≤24.04	Yes	

a Results of read mapping and binning for the WGS of one-cell RAGE-Seq reactions are shown (Materials and Methods). Each RAGE-Seq run was in three biological replicates. (A) Experiment A (in triplicates of A-1, A-2, and A-3) tests the specificity of RAGE-Seq in sorting (primarily via cellular morphology) and sequencing in a one-cell-one-tube manner (Fig. 1A). Among the three *Sc* cells showing positive MDA results, one failed in WGS sequencing library construction; thus, only two cells yield WGS results. (B) Experiment B (in triplicates of B-1, B-2, and B-3) tested the specificity of sorting and sequencing metabolically active cells (i.e., those carrying C-D peak in SCRS; Fig. 1B). (C) Experiment C (in triplicates of C-1, C-2, and C-3) tested the specificity of sorting and sequencing carotenoid-producing cells (Fig. 1C). NC, the empty droplets derived from the aqueous phase around the target cells, which served as the negative controls. %Hit_no_genomes, no hits in the NR database. Boldface indicates recovered WGS reads that match the originally targeted genome.

10.1128/mSystems.00181-21.1 FIG S1 Cells from a mock microbiota consisting of E. coli K-12 DH5α, H. pylori ATCC 26695, *S. elongatus* PCC7942, and S. cerevisiae BY4742 under a bright-field microscope. The field of view in a RAGE-Chip (A) or on a dry CaF_2_ chip (B) was shown. Red circle, S. cerevisiae BY4742 cells; blue triangle, H. pylori ATCC 26695 cells; green oval, E. coli K-12 DH5α cells or *S. elongatus* PCC7942 cells (they cannot be reliably distinguished under the microscope). (C) The workflow of Raman-activated gravity-driven cell encapsulation (RAGE). Download FIG S1, TIF file, 2.4 MB. Copyright © 2021 Jing et al. 2021 Jing et al. https://creativecommons.org/licenses/by/4.0/ This content is distributed under the terms of the Creative Commons Attribution 4.0 International license.

Specifically, experiment A (in biological replicates of A-1, A-2, and A-3) aims to test the specificity of RAGE-Seq in sorting primarily via cellular morphology. In each RAGE-Seq run, 5 cells from each of the four species (a total of 20 cells) were sorted from the mock microbiota. One-cell WGS of the corresponding MDA products suggested that 61.22% to 99.79% of the shotgun reads were mapped to the respective isolate genomes (except the 21.48% for an *Sc* cell, likely due to primer-dimer formation in its MDA reaction since 78.34% of its reads found no match in NR), with the average mapping rate for each species ranging from 52.79% to 98.83%. Among A-1, A-2, and A-3, the success rate for a given species ranged from 20% to 80%, with an average of ∼43.3% (26 MDA-positive cells in 60 sorted cells). Moreover, genome completeness of the single-cell amplified bacterial genome (SAG) eventually produced ranged between 30.14% for an *Sc* cell and 99.66% for an *Se* cell, with the average for each species ranging from 43.36% (*Sc*) to 96.78% (*Se*) (Table 1; Fig. 2A).

Experiment B (B-1, B-2, and B-3) aims to test the specificity of sorting and sequencing metabolically active cells (i.e., those carrying C-D peak in SCRS). In each RAGE-Seq run, based solely on SCRS, 11 *Ec* cells were sorted via the presence of C-D peaks, respectively. One-cell WGS results suggested that 66.35% to 94.41% of the shotgun reads were mapped to *Ec* (except the 19.13% for a cell, likely due to formation of primer dimers in MDA as 79.75% of its reads found no match in NR), and the average mapping rate was 65.00%, 77.15%, and 84.09%, respectively. For B-1, B-2, and B3, the success rate was 36.36%, 27.27%, and 18.18%, respectively (average of 27.27%; 9 MDA-positive cells in 33 sorted cells). Moreover, genome completeness of the SAG ranged between 19.13% and 94.41%, and the average at each run was 36.30%, 74.10%, and 90.30%, respectively (Table 1; Fig. 2B).

Experiment C (C-1, C-2, and C-3) was designed to test the specificity of sorting and sequencing carotenoid-producing cells. In this four-species mock community, only *Se* contains carotenoids. Thus, in each RAGE-Seq run, based solely on SCRS, 11 *Se* cells were sorted based on the presence of the carotenoid-specific peaks, respectively. One-cell WGS results suggested that 69.78% to 98.27% of the shotgun reads were mapped to *Se* (except the 17.49% for a cell, likely due to primer-dimer formation in MDA since 82.12% of its reads found no match in NR), and the average mapping rate was 74.58%, 84.16%, and 88.10%, respectively. The success rate of C-1, C-2, and C-3 was 36.36%, 27.27%, and 27.27%, with an overall success rate of 30.30% (10 MDA-positive cells in 33 sorted cells). Moreover, genome completeness of the SAG ranged between 53.80% and 99.73%, and the average at each run was 88.24%, 97.23%, and 93.30%, respectively (Table 1; Fig. 2C).

Notably, in these experiments, the empty droplets derived from the aqueous phase around the target cells, which served as the negative controls, were all free of contaminating reads from members of the mock microbiota (for them, percentage of WGS reads in the “%Hit_no_genomes” category reaches 99.53% for experiment A, 99.95% for experiment B, and 98.23% for experiment C, consistent with nonspecific amplification in MDA; Table 1). This supports the stringency of the workflow and the aqueous sorting microenvironment of RAGE-Seq, i.e., being largely free of contaminating DNA from air, surface, or reagents (which are often encountered in single-cell isolating and sequencing) (36, 37). On the other hand, in the target-cell samples, slight contamination originating from members of the mock microbiota was observed (mostly <0.5% and maximally 1.33% among all reactions in the three series of experiments; Table 1). These results suggest that the presence of DNA fragments in the liquid environment or sticking to the cell surface (which were then sorted together with target cell into the picoliter-level microdroplet by RAGE) may underlie the slight contamination observed in these one-cell RAGE-Seq reactions.

### The experimental procedure of one-cell RAGE-Seq for soil microbiota.

**FIG 3:**
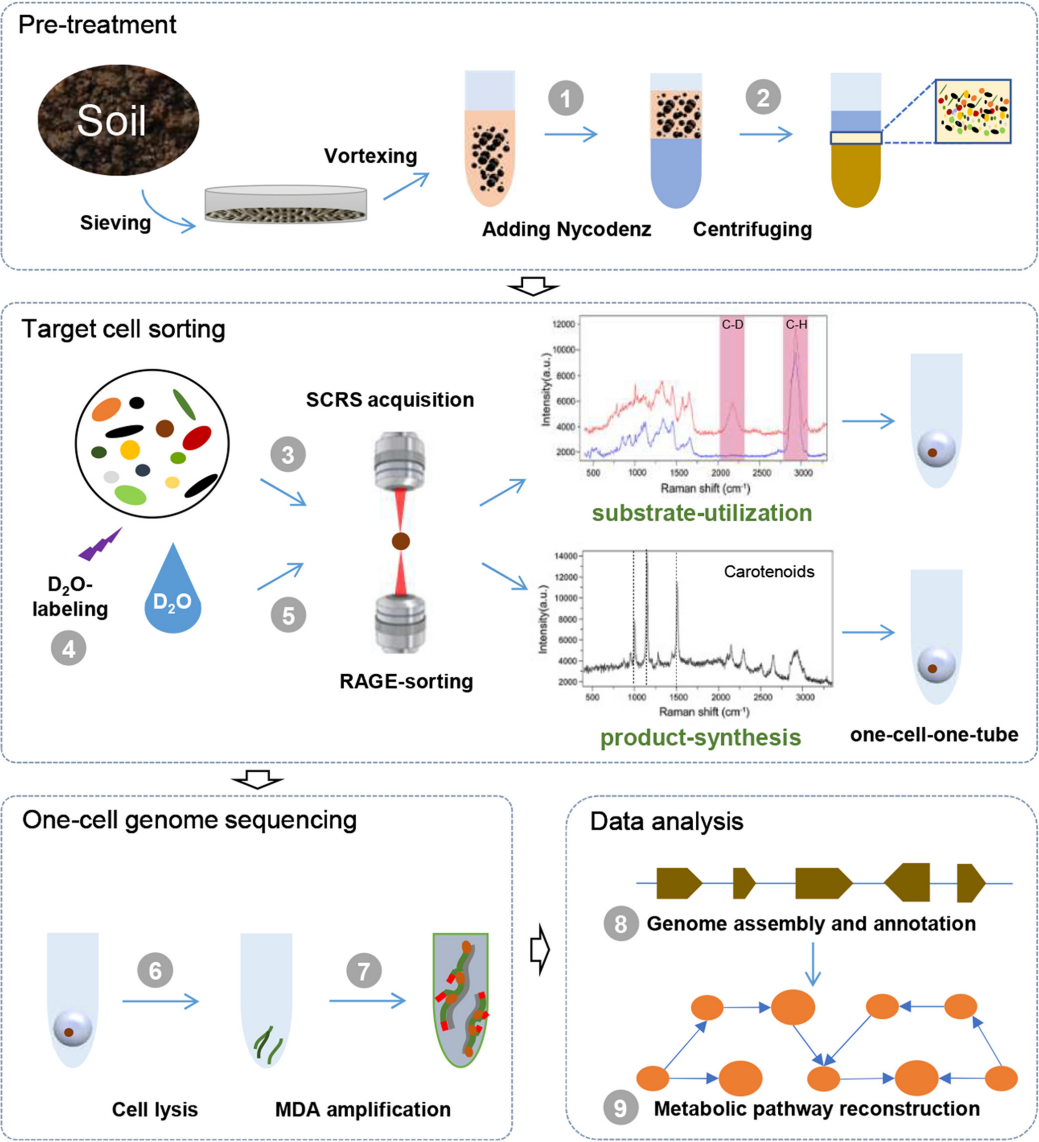
The pipeline of Raman-activated gravity-driven cell encapsulation and sequencing (RAGE-Seq) that links genotype to phenotype (metabolic activity and carotenoid production) in the soil. ① Nycodenz iohexol was added to the soil slurries. ② Microbial cells from the soil were extracted by Nycodenz density gradient separation (NDGS). ③ Carotenoid-producing cells were identified based on the characteristic Raman peak of carotenoids in SCRS and then sorted out in the RAGE chip, as a one-cell-encapsulated droplet, in a one-cell-one-tube manner. ④ For sorting metabolically active soil cells, the soil extract was incubated in 50% D_2_O for 24 h. ⑤ Metabolically active cells were identified based on the C-D band in SCRS and then sorted out as a one-cell-encapsulated droplet via RAGE. ⑥ The RAGE-sorted cells were lysed. ⑦ The genomic DNA was amplified by MDA and then processed for 16S rRNA sequencing and whole-genome shotgun sequencing. ⑧ Assembly and annotation of the single-cell shotgun sequencing reads. ⑨ Metabolic pathways were reconstructed so as to establish the link between genotype (sequencing based) and the metabolic phenotype (SCRS based) at precisely one-cell resolution.

Next, we developed a one-cell RAGE-Seq workflow for soil microbiota, which are much more diverse and complex (Fig. 3). Soil samples were collected from grassland at a depth of <3 cm on the campus of Qingdao Institute of Bioenergy and Bioprocess Technology, Chinese Academy of Sciences, China (36°9′19′′N, 120°28′50′′E). The samples were homogenized and passed through a 0.6-mm sieve to remove small stones, grass roots, and other debris. Cells in the soil were extracted using Nycodenz density gradient centrifugation (Materials and Methods). Then, in a RAGE chip (Fig. S1C), the cells were trapped and analyzed individually in a RAGE chip with a 532-nm laser, which generates SCRS with a high signal-to-noise ratio. Then, the target cell with characteristic SCRS that correspond to specific metabolic phenotypes (e.g., metabolically active cells or carotenoid-containing cells; details below) was trapped and moved with a 1,064-nm laser to form a one-cell-encapsulated droplet (Movie S1). The one-cell droplet can be readily transferred to a tube for MDA and sequencing. Notably, as the RAGE-derived one-cell droplet already carries an oil phase (mineral oil), an emulsion reaction for MDA would be formed simply via vortex in the tube (after the aqueous phase of the lysis buffer is introduced). The one-cell MDA products, after quality assessment, are then shotgun sequenced separately via HiSeq, followed by *de novo* assembly and *in silico* metabolic reconstruction for the cell, therefore linking the genome to its SCRS-derived metabolic phenotype (Fig. 3).

10.1128/mSystems.00181-21.9 MOVIE S1 Single-cell sorting of a soil microbiota sample using the RAGE chip. Download Movie S1, AVI file, 2.9 MB. Copyright © 2021 Jing et al. 2021 Jing et al. https://creativecommons.org/licenses/by/4.0/ This content is distributed under the terms of the Creative Commons Attribution 4.0 International license.

### One-cell RAGE-Seq of metabolically active bacteria in soil cell extracts via D_2_O-probed SCRS. (i) On-demand sorting and retrieval.

**FIG 4:**
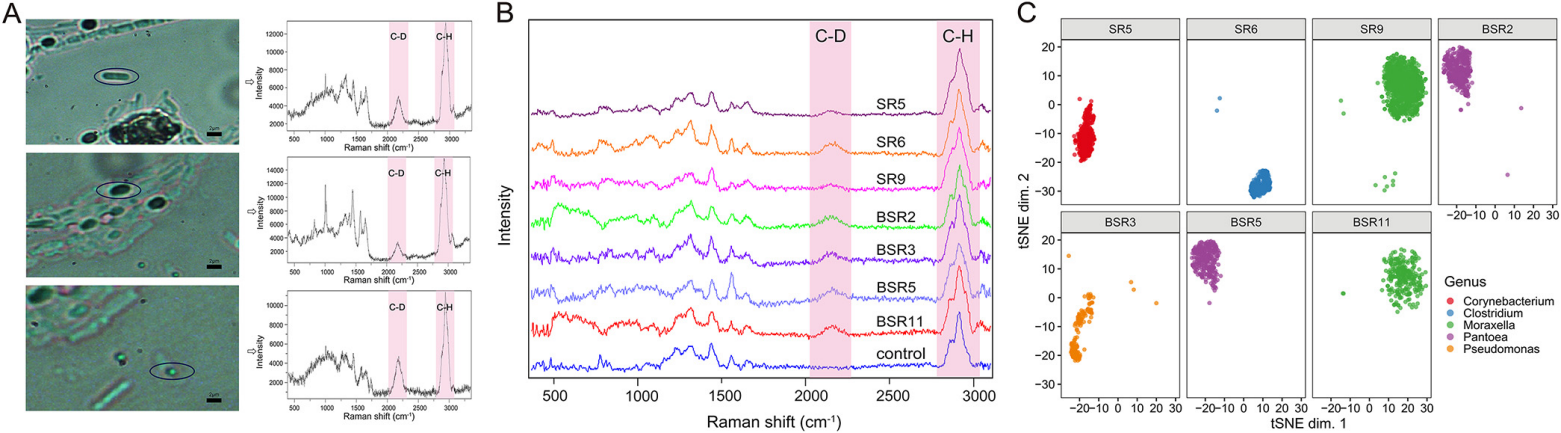
One-cell RAGE-Seq of metabolically active microbial cells from soil. (A) Various metabolically active microbes identified in the soil labeled by heavy water (D_2_O) and their corresponding Raman spectra. (B) SCRS of C-D-band-containing cells, which were treated with D_2_O for 24 h and sorted via RAGE-Seq for single-cell genomes; SCRS of SR5, SR6, SR9, BSR2, BSR3, BSR5, and BSR11, cells with C-D band; SCRS of control, cells without C-D band. (C) The t-SNE projection of binned contigs from post-RAGE single-cell sequencing reveals the taxonomical origin for the C-D-band-containing cells. Contigs are visualized based on 4-mer frequency features. Each contig is colored based on its taxonomic annotation (here the family-level annotation was shown).

To gauge the ability of RAGE-Seq to tackle substrate-utilizing phenotypes of soil microbes, extracted cells from soil were incubated with 50% D_2_O. To determine the sampling time point for RAGE-Seq, time course D_2_O-probed experiments using the soil extracts were performed (with three biological replicates; Fig. S2). At 6 h, 12 h, 18 h, and 24 h after starting the D_2_O incubation, aliquots were taken, respectively, and SCRS from 100 randomly selected cells from each of aliquots were recorded. The results revealed the gradual increase of CDR (C-D ratio) at the consortium level over time, which plateaued before 24 h (Fig. S2). Therefore, 24 h of D_2_O incubation was chosen for RAGE-Seq of the soil cell extracts. A broad Raman band appeared in the region between 2,040 and 2,300 cm^−1^, peaked at 2,157 cm^−1^ (Fig. 4A), which is the C-D stretching vibrations shifted from the C-H stretching vibrations at 2,800 to 3,200 cm^−1^ (18). This shift was attributed to the incorporation of deuterium from D_2_O to bacterial biomass via NADPH-mediated H/D exchange reactions in the metabolically active bacteria. Cells with the specific C-D bands in SCRS were then processed via RAGE-Seq, one cell per tube (Fig. 4B; one cell-free sample was taken as a negative control in each batch of experiments). To validate successful MDA for each RAGE-derived cell, the 16S rRNA gene was amplified by PCR using the MDA product as the template (Fig. S3). Nine one-cell MDA products each with clear MDA bands and positive 16S rRNA PCR results were chosen for subsequent 16S and whole-genome sequencing (Fig. S3A and B), generating ∼3 Gb of raw sequencing data for each of seven cells (SR5, SR6, SR9, BSR2, BSR3, BSR5, and BSR11; the other two failed to yield sequencing library due to severe degradation; Table 2).

**TABLE 2:** Predicted genome completeness and 16S rRNA genes of RAGE-sorted bacteria in the soil sample^*a*^

RAGE-sorted samples	Class (genus)	Estimated genome completeness (%)	Genome recovered (kbp)	GC%	Relative abundance (%) via 16S (the genus level; S1/S2/S3)	Reference(s) supporting presence of carotenoids
C-D peak-containing cells						
SR5	*Corynebacterium*	89.05	3,003.41	60.12	0/0/0.02	
SR6	*Clostridium*	22.25	1,272.90	30.05	0.01/0.02/0.03	
SR9	*Moraxella*	92.62	4,772.96	43.21	0/0/0.01	
BSR2	*Pantoea*	46.90	2,397.74	54.60		
BSR3	Pseudomonas	11.68	863.39	62.17		
BSR5	*Pantoea*	23.43	1,303.76	55.61		
BSR11	*Moraxella*	90.23	2,465.43	43.60	0/0/0.01	
Carotenoid-producing cells						
CRG1	*Pantoea*	58.66	3,419.94	52.55		46, 47
CRG2	*Legionella*	34.99	960.72	39.05	0.08/0.12/0.15	29
CRG4	*Legionella*	48.39	1,202.08	37.93	0.08/0.12/0.15	29
CRG5	*Legionella*	12.23	143.52	38.89	0.08/0.12/0.15	29
CRG6	*Massilia*	19.44	605.58	62.84	0.68/0.66/0.80	79
CRG7	Pseudomonas	20.85	1,698.06	58.72		80
CRG11	*Pedobacter*	13.01	43.11	41.25	0.05/0.05/0.06	81

a The empty droplets (NCD1 and NCD2, Fig. S3) derived from the aqueous phase, which served as the negative controls, did not contain any specific species information and were a nonspecific amplification product (accession numbers: SRR12829273 for NCD1 and SRR12829272 for NCD2). This supports the stringency of the workflow and the aqueous sorting microenvironment of RAGE-Seq.

10.1128/mSystems.00181-21.2 FIG S2 Temporal change of CDR in the soil cell extracts incubated with D_2_O over three biological replicates. (A) Patterns of mean CDR for the three replicates, with Pearson correlation coefficient of 1.00, 0.96, and 0.97, respectively. (B to E) Change of CDR distribution, for the SCRS combined from the three biological replicates (B) and for the SCRS from each of the replicates (C to E). In each of the time points for each of the biological replicates, 100 individual cells were randomly selected for SCRS acquisition. A biological replicate is a soil sample collected which, at each of the four time points, underwent an independent process of soil cell extraction, D_2_O incubation, and SCRS acquisition. CDR = area(C-D)/(area(C-D) + area(C-H)). Download FIG S2, TIF file, 0.4 MB. Copyright © 2021 Jing et al. 2021 Jing et al. https://creativecommons.org/licenses/by/4.0/ This content is distributed under the terms of the Creative Commons Attribution 4.0 International license.

10.1128/mSystems.00181-21.3 FIG S3 Agarose gel images of the multiple displacement amplifications (MDAs) and 16S rRNA gene validation processes for the soil sample. MDA products and PCR products of the 16S rRNA gene from the first batch of C-D-peak-containing cells (A), the second batch of C-D-peak-containing cells (B), and the carotenoid-producing cells (C) were shown. Lane N1, NCD1 and NCD2, empty droplets (i.e., without cells) were sorted; lane N, negative control for PCR (i.e., without adding template); lane P, positive control for PCR. Download FIG S3, TIF file, 1.9 MB. Copyright © 2021 Jing et al. 2021 Jing et al. https://creativecommons.org/licenses/by/4.0/ This content is distributed under the terms of the Creative Commons Attribution 4.0 International license.

### (ii) Recovery of high-coverage one-cell draft genomes.

After quality control, clean reads from each cell proceeded to *de novo* genome assembly (Table S1). For each cell, GC% of the assembled contigs (>200 bp; after decontamination; Materials and Methods) exhibits a normal distribution (Fig. S4A). Moreover, t-SNE (t-distributed Stochastic Neighbor Embedding) projection of contigs from each cell (>1,500 bp) via their 4-mer signatures reveals distinct clustering patterns that are characteristic to the respective taxa of the cells (Fig. 4C). These results support the accuracy of draft genome reconstruction from one-cell assemblies from RAGE-Seq.

10.1128/mSystems.00181-21.4 FIG S4 GC distributions of the contigs from one-cell SAG of RAGE-sorted, metabolically active bacteria (A) and carotenoid-producing bacteria (B) in soil. Black curves represent GC distribution of recovered draft genomes. A sliding window of 200 bp along each contig was used to extract sequence fragments and then calculate GC contents. Red curves show theoretical normal distribution with similar mean and standard deviation as the corresponding GC distribution. The GC contents of these sets of contigs exhibit normal distribution, supporting integrity of the one-cell assemblies. Download FIG S4, TIF file, 0.7 MB. Copyright © 2021 Jing et al. 2021 Jing et al. https://creativecommons.org/licenses/by/4.0/ This content is distributed under the terms of the Creative Commons Attribution 4.0 International license.

10.1128/mSystems.00181-21.6 TABLE S1 Sequencing and assembly statistics for single-cell genomes produced by RAGE-Seq. Download Table S1, DOCX file, 0.03 MB. Copyright © 2021 Jing et al. 2021 Jing et al. https://creativecommons.org/licenses/by/4.0/ This content is distributed under the terms of the Creative Commons Attribution 4.0 International license.

The identity of the target cell was determined based on that of the top contig bin (which consists of contigs binned to the same taxonomical unit). Based on DNA sequence similarity of contigs, the seven C-D-peak-present cells were pinpointed as being from *Corynebacterium* spp., *Clostridium* spp., *Moraxella* spp., *Pantoea* spp., or Pseudomonas spp. (Table 2). Interestingly, comparison of the results with 16S rRNA gene sequencing of the soil sample revealed that all of the seven cells were from low-abundance operational taxonomic units (OTUs) of the soil (all <1‰ at the OTU level; Table 2; Data Set S1).

10.1128/mSystems.00181-21.8 DATA SET S1 List of OTU abundance for the soil samples (S1, S2, and S3 represent three biological replicates). Download Data Set S1, XLSX file, 0.2 MB. Copyright © 2021 Jing et al. 2021 Jing et al. https://creativecommons.org/licenses/by/4.0/ This content is distributed under the terms of the Creative Commons Attribution 4.0 International license.

To evaluate the completeness of reconstructed one-cell genomes, CheckM (38), which is based on lineage-specific marker genes, was employed. It revealed that 89.05%, 22.25%, 46.90%, 11.68%, and 24.43% genome fractions were recovered for SR5, SR6, BSR2, BSR3, and BSR5, respectively (Table 2). The contigs in SR9 and BSR11, both assigned to *Moraxella* spp., represent 92.62% and 90.23% completeness of their genomes, respectively (Fig. 4C; Table 2). This supports the feasibility of RAGE-Seq to produce high-coverage genomes from just one post-RACS soil bacterial cell.

Moreover, no significant difference in either completeness or GC content is apparent between the seven post-RAGE one-cell genomes and the one-cell genomes from the 17 randomly chosen, droplet-packaged bacterial cells from single-droplet MDA (sd-MDA [39]; Wilcoxon test; *P* > 0.05; Fig. S5A and B). Thus, in RAGE-Seq, the SCRS acquisition process (i.e., for metabolic phenotyping) does not seem to hamper subsequent one-cell MDA and sequencing, which is in sharp contrast to RACE-Seq, when the laser exposure during SCRS acquisition is the most important factor that negatively impacts the quality of post-RACE MDA and sequencing (29).

10.1128/mSystems.00181-21.5 FIG S5 Performance comparison between RAGE-Seq (one cell per reaction) and sd-MDA (39) method. For RAGE-Seq, each of seven C-D-peak-containing cells (SR5, SR6, SR9, BSR2, BSR3, BSR5, and BSR11) was individually sorted from soil and separately sequenced (from precisely one cell). For sd-MDA, 17 bacterial cells were randomly isolated from soil samples and underwent single-cell sequencing using droplet microfluidics. (A) Completeness of the SAGs based on the evaluation using marker genes; (B) GC% of obtained draft genomes. Download FIG S5, TIF file, 0.5 MB. Copyright © 2021 Jing et al. 2021 Jing et al. https://creativecommons.org/licenses/by/4.0/ This content is distributed under the terms of the Creative Commons Attribution 4.0 International license.

### One-cell RAGE-Seq of carotenoid-producing cells in soil cell extracts via characteristic Raman peaks of carotenoids.

**FIG 5:**
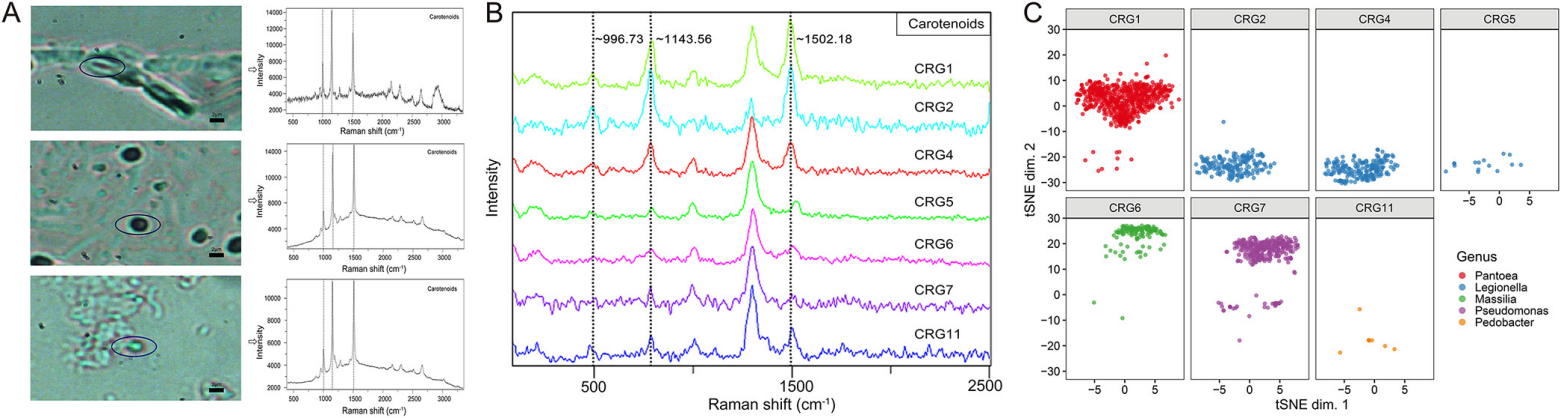
One-cell RAGE-Seq of carotenoid-producing microbial cells from soil. (A) Various carotenoid-producing microbes identified in the soil via characteristic Raman peak of carotenoids and their corresponding SCRS. (B) SCRS of carotenoid-producing cells sorted via RAGE-Seq for single-cell genomes, with sample names CRG1, CRG2, CRG4, CRG5, CRG6, CRG7, and CRG11, respectively. (C) The t-SNE projection of binned contigs from post-RAGE single-cell sequencing reveals the taxonomical origin for the carotenoid-producing cells. Contigs are visualized based on 4-mer frequency features. Each contig is colored based on its taxonomic annotation (here the family-level annotation was shown).

To gauge the ability of RAGE-Seq to tackle product synthesis-related phenotypes, which are of interest for bioresource mining from soil, we focused on carotenoids, one of the most diverse classes of pigments (with over 700 kinds found [40]) and found in nearly all photosynthetic cells to protect them from exposure to UVA radiation (41). They also give characteristic bands in SCRS in 1,500 to 1,550, 1,150 to 1,170, and 1,000 to 1,020 cm^−1^ (as caused by in-phase C=C [*v*3], C–C stretching [*v*2] vibrations of the polyene chain, and in-plane rocking mode of CH_3_ groups attached to the polyene chain [*v*1] [15, 22, 34, 35]), which can serve as distinct criteria for RACS (22, 23). In our soil samples, the carotenoid-producing cells showed the characteristic Raman peak of *v*1, *v*2, and *v*3, as expected (Fig. 5A).

Here, similar to sorting metabolically active cells, individual carotenoid-producing cells were isolated based on the characteristic peaks via RAGE from the same soil samples and then sequenced (Fig. 5B and Fig. S3C). One-cell genome assemblies of the seven such cells (numbered CRG1, CRG2, CRG4, CRG5, CRG6, CRG7, and CRG11) pinpoint them as from *Pantoea* spp., *Legionella* spp., *Massilia* spp., Pseudomonas spp., and *Pedobacter* spp., respectively (Table 2 and Table S1). GC% of the assembled contigs (>200 bp; after decontamination; Materials and Methods) exhibits normal distribution (Fig. S4B). In addition, the contigs of each cell show distinct clustering patterns characteristic of the respective taxa of the cells, based on t-SNE projection via their 4-mer signatures (Fig. 5C). These results support the integrity of the one-cell genome reconstructions. Notably, of the seven cells, three were of low-abundance bacterial genera, as suggested by relative abundance of 16S rRNA gene (<1‰; Table 2). Moreover, members of the five phyla were known to synthesize carotenoids (Table 2), which is consistent with RAGE sorting criteria.

### (i) Recovery of high coverage one-cell draft genomes.

**FIG 6:**
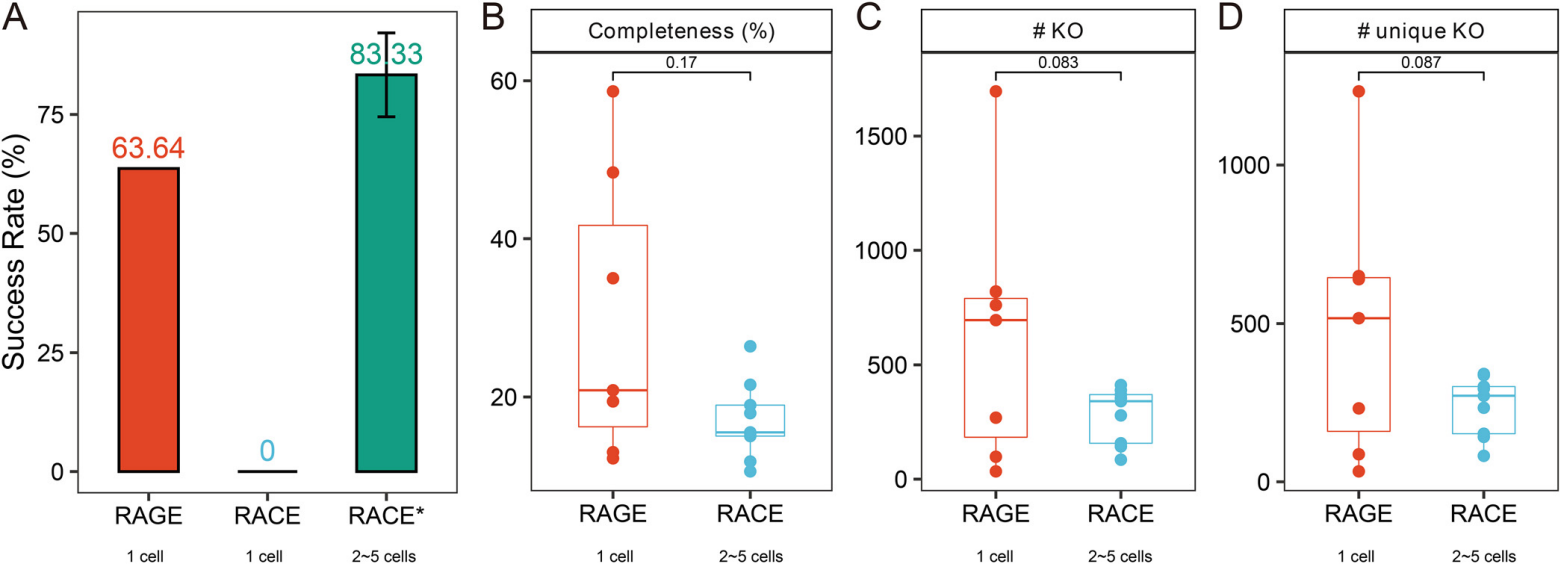
Performance comparison between RAGE-Seq (one cell per reaction) and RACE-Seq (2 to 5 cells per reaction). For RAGE-Seq, each of seven carotenoid-containing cells (CRG1, CRG2, CRG4, CRG5, CRG6, CRG7, and CRG11) was individually sorted from soil and separately sequenced (from precisely one cell). For RACE-Seq, individual carotenoid-containing cells were ejected separately. However, one-cell sequencing of RACE-Seq suffered from a very low success rate (and has not succeeded in past works); thus, the post-RACE cells were then merged into seven pools (C2, C4, X18, X1, X17, X6, and R4), each containing 2 to 5 cells, prior to the MDA reaction. (A) Comparison of success rates in 16S rRNA gene PCR for the RAGE-sorted soil cells and RACE-sorted soil cells (RACE for single cell, RACE* for 2- to 5-cell pools). (B) Completeness of the SAGs based on the evaluation using marker genes. (C and D) Functional elements mined from the *de novo* assemblies: KEGG ontologies (C) and unique KOs (D).

The overall success rate of one-cell RAGE-Seq (i.e., number of successful experiments divided by total number of attempts, with “success” defined as the ability to produce from the MDA product the target 16S-rRNA via PCR and then verify by sequencing), at 63.64%, is much higher than RACE-Seq (no success for one-cell reactions) (29) (Fig. 6A). To evaluate the completeness of reconstructed one-cell genomes, CheckM (38), which is based on lineage-specific marker genes, was employed, revealing estimated genome completeness of 12.23% to 58.66% (average of 29.65%, Table 2). Even though each of them is from just one carotenoid-producing bacterial soil cell, genome completeness values of post-RAGE-Seq cells are equivalent to those of RACE-Seq (each as a pool of 2 to 5 soil bacterial carotenoid-producing bacterial cells; 17.03%; *P* = 0.17; Wilcoxon test; Fig. 6B). In particular, one-cell genome completeness via RAGE-Seq can reach 58.66% (for *Pantoea* spp.), which is much higher than the best case for the optimized RACE-Seq, where completeness from 2 to 5 carotenoid-producing soil cells was just 26.42% (one-cell MDA reactions from RACE-Seq all failed for the soil samples) (29) (Table 2). As a result, many more functional genes can be unraveled through RAGE-Seq than RACE-Seq, as evidenced by average number of KO (KEGG Orthology, 625 versus 281; *P* = 0.083; one-sided *t* test; Fig. 6C) and unique KO (484 versus 239; *P* = 0.087; one-sided *t* test; Fig. 6D).

### (ii) Reconstructing carotenoid-biosynthetic pathways from RAGE-Seq-derived one-cell assemblies.

From contigs (>200 bp) of each of the seven RAGE-Seq-derived one-cell assemblies (CRG1, CRG2, CRG4, CRG5, CRG6, CRG7, and CRG11), we reconstructed the carotenoid-biosynthetic pathway by mapping the predicted gene sets to the reference KEGG metabolic pathway (KO00906 and KO00900 [42]) based on sequence homology. As a control, contigs of each of seven 2- to 5-cell-pooled samples derived from an optimized RACE-Seq protocol (C2, C4, X18, X1, X17, X6, and R4) (29) underwent an identical computational procedure for the pathway reconstruction.

**FIG 7:**
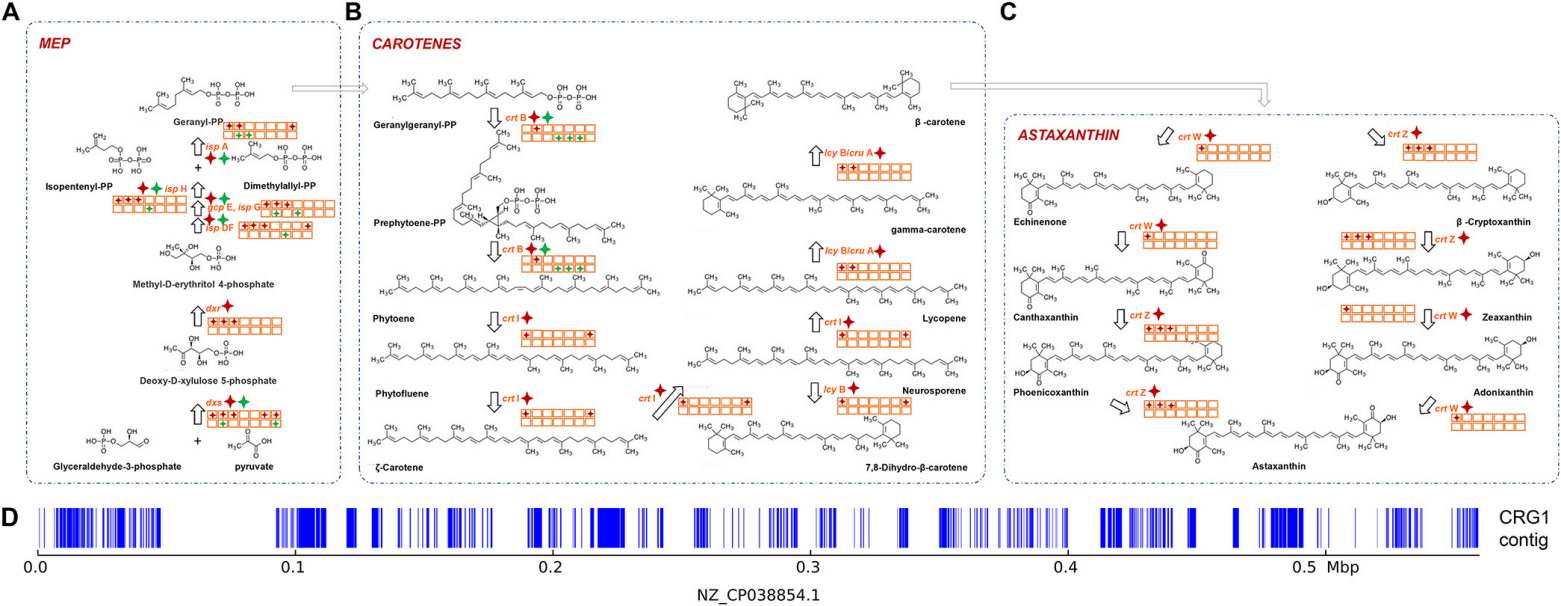
Comparison of the reconstructed carotenoid synthesis pathway between RAGE-Seq (one cell per reaction) or RACE-Seq (2 to 5 cells per reaction) from soil microbiome. (A to C) The modules of MEP (A), β-carotene (B), and astaxanthin (C) biosynthesis are shown. The seven RAGE-Seq-derived sequence assemblies (1 cell per MDA reaction) are indicated as squares in the top row (left to right: CRG1, CRG2, CRG4, CRG5, CRG6, CRG7, and CRG11), while the seven RACE-Seq-derived ones (2 to 5 cells per MDA reaction) are indicated as squares in the bottom row (left to right: C2, C4, X18, X1, X17, X6, and R4). Red star, found in a RAGE-Seq assembly; green star, found in a RACE-Seq assembly. (D) In CRG1, all the recovered carotenoid-synthetic genes are mapped to a plasmid from Pantoea vagans with accession no. NZ_CP038854.1, suggesting they are likely from a plasmid in the hosting cell. Those sequence blocks in each contig that are aligned to NZ_CP038854.1 are shown as blue lines.

The biosynthetic pathways for carotenoids are widely found in plants and bacteria (43). Like all isoprenoids, carotenoids are synthesized from the production of isopentenyl pyrophosphate (IPP), which primarily occurs through the methyl d-erythritol 4-phosphate (MEP) module that begins with glyceraldehyde-3-phosphate (Fig. 7A). Then, from four IPP molecules geranylgeranyl diphosphate (GGPP), which contains 20 carbon atoms, is synthesized. By condensing two GGPPs, the first carotene precursor, phytoene, is produced by phytoene synthase. Phytoene is converted to lycopene in four desaturation steps, and lycopene is then cyclized on both ends to form β-carotene (Fig. 7B). Then, identical hydroxylation of both β-carotene rings yields zeaxanthin, which can be epoxidated to form antheraxanthin or violaxanthin. Neoxanthin and other pigments are derived from violaxanthin, zeaxanthin, or β-carotene from which, e.g., astaxanthin is derived. In addition to the reconstructed MEP and β-carotene modules, our data reveal that the synthetic pathways of astaxanthin, a reddish keto-carotenoid classified as a xanthophyll found in various microbes (44), can also be reconstructed (Fig. 7C).

Interestingly, in CRG1, which was assigned to *Pantoea* spp. (a member of the *Enterobacteriaceae* family), the contigs from CRG1 were mapped to 0.19-Mb sequences (33.16%) and 145 genes of a plasmid from *Pantoea* (accession number NZ_CP038854.1; of 0.56 Mb and harboring 516 genes in total) (Fig. 7D); this suggests the ability to (at least partially) reconstruct mobile elements from one-cell RAGE-Seq assemblies. In addition, many genes in the β-carotene and astaxanthin modules were found from the sequences aligned to NZ_CP038854.1, suggesting their plasmid origin in CRG1. Notably, *Pantoea* spp., due to their rich production of carotenoids, have served as a model organism to provide pigment synthesis components (45–47): e.g., five biosynthetic genes in the natural carotenoid cluster from the cultured Pantoea ananatis were cloned and used to optimize zeaxanthin production in E. coli (48). Therefore, from just one yet-to-be-cultured bacterial cell recognized as carotenoid producing (via its SCRS), RAGE-Seq can recover the underlying biosynthetic genes, representing a new route to mine functional genes and pathways in a function-based yet culture-free manner.

The one-cell RAGE-Seq assemblies support more complete and more in-depth discovery of carotenoid-synthetic genes and pathways than 2- to 5-cell-pooled RACE-Seq assemblies. (i) In the MEP module, all the enzymes were identified in the data set merged from the seven RAGE-Seq-derived samples. Notably, each of CRG1 (*Pantoea* spp.), CRG2 (*Legionella* spp.), and CRG4 (*Legionella* spp.), the ones with top estimated genome completeness (58.66%, 34.99%, and 48.39%, respectively), was able to reconstruct by itself the complete MEP module (Fig. 7A), with the exception of the *isp*A gene in CRG4. In contrast, although each enzyme was found by at least one sample (except *dxr*), none of the seven RACE-Seq samples alone can reconstruct the complete MEP module (Fig. 7A).

(ii) In the β-carotene module, the first step unique to carotenoid synthesis is the synthesis of geranylgeranyl diphosphate (GGPP) via the enzyme GGPP synthase. Then it forms the phytoene, which is the precursor of all carotenoids. This reaction, catalyzed by the phytoene synthase (*crt*B), is considered the main bottleneck in the carotenoid pathway (49). However, only CRG2 from RAGE-Seq reports this enzyme (Fig. 7B). The CRG1 sample from RAGE-Seq recovered *crt*I and *lcy*B enzymes (but no *crt*B enzyme). Genes encoding all steps for carotenoid formation from GGPP were identified in collective one-cell assemblies from RAGE-Seq (Fig. 7B), and the most complete pathway reconstructed was also in CRG1 (Fig. 7B). In contrast, RACE-Seq recovered *crt*B in only three of the samples, and all the other enzymes in this module are missing in each sample (Fig. 7B).

(iii) In the astaxanthin module, *crt*W encodes β-carotene ketolase, which is one of the key enzymes in astaxanthin biosynthesis that catalyzes the formation of canthaxanthin from β-carotene via echinenone, while the *crt*Z gene encodes β-carotene hydroxylase. For RAGE-Seq, CRG1 reports two astaxanthin biosynthetic genes of *crt*W and *crt*Z, and both CRG2 and CRG4 report the *crt*Z gene (Fig. 7C). However, RACE-Seq missed all the genes in this module (Fig. 7C).

Notably, cultured relatives of the individual cells characterized via RAGE-Seq were potentially able to synthesize carotenoids (Table 2). Together with the SCRS-based cell sorting criterion, these items of evidence support the ability of RAGE-Seq to link the carotenoid-synthetic phenotype with the underlying genomes at precisely one-cell resolution from soil.

### Links between the D_2_O intake and carotenoid-producing phenotypes among soil cells.

**FIG 8:**
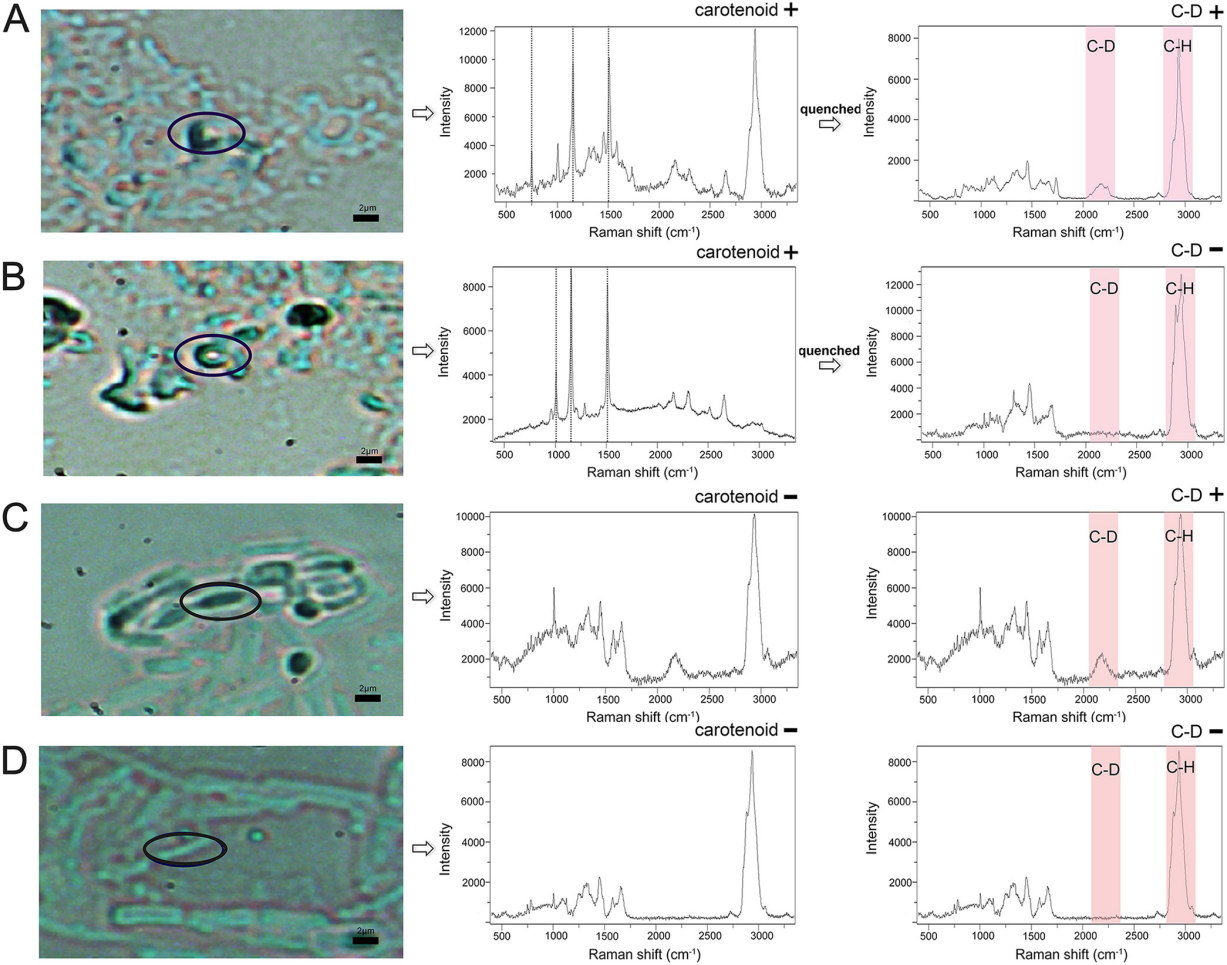
Links between the D_2_O intake and carotenoid-producing phenotypes among individual soil cells. (A) A carotenoid-producing cell whose SCRS harbors the C-D peak after the carotenoid peaks were quenched. (B) A carotenoid-producing cell whose SCRS does not harbor the C-D peak after the carotenoid peaks were quenched. (C) A cell whose SCRS harbors the C-D peak but does not show carotenoid peaks (i.e., not producing carotenoids). (D) A cell whose SCRS shows neither the C-D peak nor the carotenoid peaks.

Interestingly, in the soil cell extracts, carotenoid-producing cells as recognized by their pigment spectra can either have C-D peaks (metabolically active, Fig. 8A) or not (metabolically inert, Fig. 8B). Thus, it is likely that many soil microbes that produce carotenoids might be metabolically dormant. This also suggests that cultivation-based efforts that depend on metabolically active cells can miss many potential microbial cell factories of carotenoids.

On the other hand, for those not showing carotenoid peaks in SCRS, both cells with C-D peaks (Fig. 8C, suggesting active metabolism) and cells without C-D peaks (Fig. 8D, inactive metabolism or dead cells) were found. Among the over 1,200 soil cells analyzed for SCRS in the replicated time course experiments, no correlation at the single-cell level is apparent between carotenoid-synthetic phenotype and the phenotype of general metabolic activity (i.e., D_2_O incorporation rate). This suggests the importance of the single-cell phenome, i.e., simultaneous profiling of multiple phenotypes for a given cell, in fully reconstructing “who is doing what” in soil.

## DISCUSSION

For microbiomes that are functionally and genetically as diverse and heterogeneous as soil, the phenotype-dedicated one-cell genome sequencing strategy of RAGE-Seq is of particular importance to mechanistic dissection and functional-gene mining, where both numerically abundant and rare taxa can play important roles in microbiome activity. In contrast, in metagenomic sequencing of soil, reconstructing the genomes of functionally important members can be difficult, where sequence reads from numerically abundant species would dominate and, moreover, the difficulty in read binning hinders reliable genome assembly. Moreover, the ability to link genome sequence to an anabolic or catabolic activity (e.g., D_2_O assimilation or carotenoid synthesis) at precisely one-cell resolution is a pivotal advantage, since it provides one direct answer to the ultimate question of “Who is doing what? Why?” for the microbiome.

Like RAGE-Seq, RACE-Seq (which ejects a cell from a solid surface) can also phenotype, sort, and sequence cells in an “index” manner (30), yet applications of RACE-Seq to microbiomes from seawater (22, 23), soil (29), and humans (20) are limited by the inability to produce high-quality genome sequence at the resolution of one cell (see Table S2 in the supplemental material), likely due to the reduction or loss of cell vitality when cells are air dried at a solid surface prior to Raman exposure, the direct exposure of the cell to the Raman exciting laser, and the effect of the pulsed laser for cell ejection on the cell (29). Specifically, in RACE-Seq, for DH5α cells, the highest genome coverage for five-cell pooled MDA reactions (after our optimization) was only 60%, and for soil microbiome, the genome completeness was no more than 30% for 2- to 5-cell pooled MDA reactions. As for precise one-cell reactions, the success rate of RACE-Seq has been very low even for pure cultured E. coli (<10%), while for soil microbiome all one-cell reactions via RACE-Seq have failed (29). In contrast, our results on soil microbiota here showed that precisely one-cell RAGE-Seq of diverse soil microbes can produce genome coverage as high as ∼93%. The ability of RAGE-Seq to support phenotyping-sorting-sequencing at the resolution of one bacterial cell is due to single-cell indexing via precise single-cell capture and on-demand microdroplet encapsulation and the full preservation of cell vitality via aqueous-phase Raman detection and sorting, which ensures robust, high-quality sequencing and cultivation of the post-RAGE cell by avoiding laser-induced damage (33). Therefore, RAGE-Seq is particularly suitable for reliable metabolic phenotyping and sequencing of bacteria at precisely one-cell resolution from environmental samples (Table S2).

10.1128/mSystems.00181-21.7 TABLE S2 Comparison of instruments, techniques, and their performance for Raman-activated cell sorting and genome (RACS-Seq) among studies. Download Table S2, DOCX file, 0.03 MB. Copyright © 2021 Jing et al. 2021 Jing et al. https://creativecommons.org/licenses/by/4.0/ This content is distributed under the terms of the Creative Commons Attribution 4.0 International license.

The RACS family includes additional tools that can potentially be coupled to downstream genome sequencing (14, 27). However, although the throughput of flow-mode RACS such as RAMS (Raman-activated microfluidic sorting, ∼60 cells/min for carotenoid peaks in yeast [50]), RADS (Raman-activated droplet sorting, ∼260 cells/min for astaxanthin peaks in Haematococcus pluvialis [51]), or those based on optical tweezers (3.3 to 3.8 cells/min for C-D peaks for mouse gut bacteria [21]) are higher than RAGE, they have not yet demonstrated the ability to link the sequencing-based genotype to the SCRS-derived phenotype at the one-cell level, which can be a challenge as the “index” of sorted cells is usually lost after the high-throughput flow sorting.

On the other hand, the current implementation of RAGE-Seq has several limitations. First, the overall success rate of one-cell reactions, ∼36% for the mock microbiota (45 MDA-positive cells in 126 sorted cells) and ∼41% for the soil microbiota (14 MDA-positive cells in 34 sorted cells) here, should be improved. In fact, all the published RACS-Seq studies so far have suffered from this problem (29, 30, 33). This is likely due to the radiation of Raman laser during SCRS acquisition (29). In one-cell MDA, which is another challenge (although not a problem of RACS itself), bias against high-GC-content DNA (52), hindering of reaction success by low-abundance templates (53), and negative effect due to highly fragmented contaminant DNA (54) have been reported. Thus, the overall success rate of RAGE-Seq can perhaps be elevated by reducing incident laser power and measurement time (29, 55) or by reducing the volume of MDA reactions (56, 57). Second, among the WGS reads from one-cell RAGE-Seq reactions a certain proportion of contaminating reads was present. This can be due to the environmental DNA fragments (either sticking to the cell surface or floating in the surrounding liquid setting) that were sorted together with target cell into the picoliter-level microdroplet by RAGE. Bias of MDA can magnify the proportion of such contaminating DNA, which may exacerbate the situation. Therefore, further improvement of RAGE-Seq experimental workflow will be important, such as optimizing cell incubation and washing process prior to SCRS acquisition and sorting (to reduce contaminating DNA fragments which can readily attach to cell surface) (58) and further decrease of microdroplet volume (which reduces the amount of floating-environment-DNA templates in the MDA reaction). Third, the relatively low throughput of RAGE-Seq in sorting (at 2 cells/min) and MDA (one-cell-per-tube) at present has hindered time- and cost-efficient analysis of hundreds or thousands of cells from a genetically and functionally diverse microbiome such as soil. This shortcoming, which led to the low microbiome sampling depth here, has limited the scope and nature of ecological insights that can be derived (i.e., findings from the sampling by RAGE-Seq of only a very small portion of cells in consortium should be interpreted with caution). The sorting throughput can be improved by adopting techniques such as surface-enhanced Raman scattering (SERS) or stimulated Raman scattering (SRS) which offer much higher Raman signals and thus reduce SCRS acquisition time, so that a much higher number of single cells can be efficiently profiled for their metabolic phenotypes (and genomes once sorted) from each microbiome. Flow-mode RACS methods can also potentially tackle this challenge (21, 59, 60) (Table S2), although solutions that couple the sequential SCRS-based sorting to paralleled, indexed preparation of microbial one-cell sequencing libraries remain to be developed. Finally, interpretation of findings from microbiome RAGE-Seq is also limited by, and dependent on, sample pretreatment. For example, the extraction of cells from their native microenvironment in soil prior to D_2_O feeding and Raman microspectroscopy can alter both the *in situ* metabolic activity and relative abundance of soil cells. Therefore, in addition to improving the throughput of RAGE-Seq, future efforts should include development of microbiome-feeding and cell extraction methods that maximally preserve the *in situ* population structure and metabolic activity of soil inhabitants.

Despite these challenges, the soil RAGE-Seq workflow introduced here is widely applicable, as the scope of metabolic phenotypes that can be derived from nonresonance (e.g., the C-D band) or resonance peaks (e.g., carotenoid-related bands) in SCRS is rapidly expanding (14). For example, SCRS can detect not just D_2_O intake (and phenotypes that are correlated with D_2_O intake such as antibiotic resistance) (17) but assimilatory activity of carbon or nitrogen sources labeled by stable isotopes of C or N (19, 61). Similarly, the ability to mine both the cellular carotenoids (in both abundance and structure) and the underlying genotypes encoding biosynthetic pathways at the individual cell level should allow mining for not just carotenoids but lipids, polysaccharides, protein, and even antibiotics (24, 25, 62, 63). Therefore, it is possible that RAGE-Seq would become a universal and highly versatile tool for precisely probing “who is doing what” and for mining cells or metabolites of interest, from soil and other complex natural ecosystems.

## MATERIALS AND METHODS

### Bacterial species, media, and growth conditions.

The series of mock microbiota all include the three bacteria E. coli K-12 DH5α, H. pylori ATCC 26695, and *S. elongatus* PCC7942, and the one fungus S. cerevisiae BY4742. Each of the strains was grown in pure culture. H. pylori ATCC 26695 (Qingdao Municipal Hospital, China) was cultured in brain heart infusion (BHI) agar (Oxoid, Basingstoke, England) supplemented with 7% defibrinated horse blood and incubated under microaerophilic conditions (85% N_2_, 10% CO_2_, 5% O_2_) at 37°C. H. pylori ATCC 26695 was obtained by centrifugation at 8,000 × *g* for 2 min and washed twice with BHI, and resuspended cells were diluted to an OD_600_ of ∼0.5 and inoculated at a ratio of 1:10 into 10 ml of BHI broth with 10% fetal bovine serum (FBS). E. coli K-12 DH5α was cultured in Luria-Bertani (LB) medium (tryptone, yeast extract, NaCl, pH 7.0) and incubated at 37°C. E. coli K-12 DH5α was diluted to an OD_600_ of ∼0.5 and inoculated at a ratio of 1:10 into 4 ml of LB medium. S. cerevisiae BY4742 was cultured in YPD medium (yeast extract, peptone, glucose, pH 6.5 to 6.8) and incubated at 30°C. S. cerevisiae BY4742 was diluted to an OD_600_ of ∼0.5 and inoculated at a ratio of 1:10 into 4 ml of YPD medium. *S. elongatus* PCC7942 was cultured in BG11 medium and incubated under lighting at 28°C. In these dilution experiments, we assumed a constant relationship of OD and cell concentration for all the cell types; while this might not be correct for each of them, this assumption would not change our experimental findings and interpretations. For deuterium isotope labeling, 50% (vol/vol) D_2_O (99.9 atom% D; Sigma-Aldrich, Canada) was used in all the above media. To prepare the media for deuterium isotope labeling, 2× medium was prepared with water and autoclaved and then diluted to 1× medium with filtered pure D_2_O, so that the eventual level of D_2_O was 50%. Each of the microorganisms was incubated in the respective medium containing 50% D_2_O until reaching the logarithmic phase, washed using distilled water, and mixed to form the synthetic consortia with defined structure. The synthetic consortia were then subjected to single-cell Raman spectroscopy and SCRS-based sorting, respectively.

### Benchmarking performance of the one-cell RAGE-Seq method via mock microbiota.

To semiquantitatively assess performance of the one-cell RAGE-Seq method, we performed replicated RAGE-Seq experiments on the synthetic four-species mock microbiota. Three different sorting criteria were respectively employed: criteria A (via cell morphology), B (D_2_O-peak-containing cells), and C (carotenoid-peak-containing cells), which sorted and sequenced 20 cells, 11 cells, and 11 cells, respectively, per experiment. Within each of the sorting criteria, three biological replicates were performed. Thus, on the synthetic four-species mock microbiota, nine RAGE-Seq experiments in three biological replicates were performed, which sorted and sequenced 126 individual cells in total. Method performance was then assessed via mapping rate (i.e., the number of mapped reads/total sequencing reads), success rate (i.e., the number of 16S-sequencing-validated SAGs/total sorted cells), and genome completeness (i.e., the percentage of aligned bases from assembled contigs in the reference genome; a base in the reference genome is aligned if there is at least one contig with at least one alignment to this base) of the one-cell sorting and sequencing results.

### Extracting bacteria from soil and deuterium labeling of microbial cells.

To extract the cells from soil, the soil slurries, generated by adding 1 g soil into 5 ml 1× PBS buffer supplemented with 25 μl Tween 20, were vortexed for 30 min for freeing the particle-associated cells. In a new 15-ml centrifuge tube, 5 ml Nycodenz iohexol (1.42 g/ml; Aladdin, China) was added, and then the aforementioned supernatant from soil slurries was slowly added to the top of Nycodenz. The tubes were centrifuged at 14,000 × *g* for 90 min at 4°C with slow acceleration and deceleration. At the middle layer, which is between the clear PBS layer and the debris layer, a faint whitish band containing bacterial cells would emerge (19, 64). This band was recovered and transferred into a new 1.5-ml Eppendorf tube with a pipette. Then, 1 ml double-distilled water (ddH_2_O) was added to resuspend the cells, and the cells were pelleted by centrifugation at 10,000 × *g* for 10 min at 4°C, 3 times. Finally, the cell pellets were resuspended in 0.2 ml ddH_2_O, which represented the “soil cell extracts.”

These soil cell extracts were then used for mining either carotenoid-producing cells or D_2_O-intake cells (i.e., metabolically active cells). For SCRS acquisition or RAGE-Seq of carotenoid-producing cells, the soil cell extracts were directly used. As for D_2_O-probed SCRS acquisition and RAGE-Seq experiments for metabolically active cells, the soil cell extracts were then incubated in PBS with a final D_2_O level of 50% at room temperature for a certain duration. To determine the sampling time point for RAGE-Seq of metabolically active cells, D_2_O-probed SCRS acquisition experiments were performed that temporally monitored D_2_O intake by the soil cell extracts, where aliquots were taken at 6 h, 12 h, 18 h, and 24 h, respectively, for Raman microspectroscopy. Based on the results, we chose 24 h for the RAGE-Seq experiments that target soil cells that actively assimilate D_2_O.

### Acquisition of single-cell Raman spectra.

The RAGE-Seq procedure was performed in a RACS-Seq instrument (Qingdao Single-cell Biotechnology, Qingdao, Shandong, China). Before the Raman test, the bacterial samples were washed to remove residual medium and then resuspended by adding deionized water to dilute them for performing the following Raman detection and sorting. The prepared bacterial solution (∼1 ml) was hung up on the sample holder and then loaded into the RAGE chip. Raman spectra were acquired with a modified confocal Raman microscope. A 50× dry objective (numerical aperture [NA] = 0.65; Leica, Germany) was used for sample signal acquisition and optical tweezers, while a 10× dry objective was used for observation of droplet generation and transportation. All Raman spectra were preprocessed by background noise subtraction, baseline correction, and normalization to C-H band via LabSpec5 software. Cells with a C-D band in SCRS were isolated using RAGE-chip as described above. The selection of post-SCRS-acquisition cells to be sorted was based on a computer algorithm. Specifically, the sorting was based on C-D/(C-D + C-H); this ratio was calculated via dividing C-D peak area from 2,040 to 2,300 cm^−1^ by the sum of C-D area and C-H peak area from 2,800 to 3,100 cm^−1^. The whole pipeline used here has been made available on GitHub (https://github.com/gongyh/RamanD2O). The tube which contained the target cells was then moved into a laminar hood, and buffer (Qiagen, Germantown, MD, USA) was added into the tube for cell lysis.

### Chip fabrication for Raman-activated gravity-driven cell encapsulation (RAGE).

The RAGE chip consists of two slides bound together with a semiopen design, as shown in Fig. S1 in the supplemental material, one sample inlet, and one open well for oil storage and droplet generation. For the top entered laser (laser entering from top slide), the microchannel was etched on the bottom layer slide. The channel was sealed by a smooth slide with two holes, one hole (∼0.5 mm in diameter) for the inlet and one (5 mm in diameter) for the open well. With this design, the focus point and trapping force of lasers were not affected after penetrating the top layer of the chip. The scale of the thin channel between detection window and open well was adjusted for different sizes of cells. We used the 30-μm-width and 10-μm-depth microchannel for the sorting of microbial cells in soil.

### Isolation of target cells from the soil sample by RAGE.

The cells in suspension were injected into the chip with the height-adjustable sample holder. The well was filled with mine oil (2% wt EM90) when the cell phase reached the open well. The height of the sample holder was adjusted to obtain a balance between the water phase and oil phase. The cells located statically in the detection window were trapped and analyzed with the 532-nm laser to identify target cells. The cell sorting can also be based on visible phenotypes such as morphology or autofluorescence without the 532-nm laser. Then, a second laser (1064 nm) was employed to trap and move the target cell to the tip of the aqueous phase. The sample holder was elevated to generate only one droplet that encapsulates the cell and then lowered to the original height. The single target cell was thus isolated and encapsulated in the droplet. As the density of the oil used here is lower than water, the droplet stays statically at the bottom of the open well. At the end, the droplet with the target cell can be easily taken out to a tube for downstream analysis, with a pipette tip.

Notably, the bacterial cell adsorption onto the channel wall can be neglected here in our quartz chip. Although the in-chip environment is a static mode, the majority of cells still show irregular movements during sorting due to pedesis or flagellum effect. In addition, new cells can be injected into the chip continuously by elevating the sample holder. The volume on the sample holder which is connected into the sorting chip is usually 1 to 2 ml, yet the volume of the chip is only <5 μl; thus, all the cells in the chip can be replaced hundreds of times when necessary, without the need for new loading. In our current setting, the sorting throughput can be maintained for at least 3 h, and cell settling is not our major concern.

### Multiple displacement amplification.

The REPLI-g single-cell kit (Qiagen, Hilden, Germany) was used for the DNA amplification. Cell lysis was carried out at 65°C for 15 min with 2 μl lysis buffer for each sample, followed by addition of 1 μl stop solution to neutralize the lysis buffer. REPLI-g single-cell (sc) reaction buffer and REPLI-g sc DNA polymerase were added, and the mixture was incubated at 30°C for 8 h with a 70°C hot-lid temperature for MDA reactions. Blank control (without any cells) was also included to detect and quantify potential contamination. After that, the MDA products were processed for 16S rRNA gene (with primers 27F [forward, 5′-AGAGTTTGATCCTGGCTCAG-3′] and 1492R [reverse, 5′-TACGGYTACCTTGTTACGACTT-3′]) and/or 18S rRNA gene (with primers NL1 [forward, 5′-GCATATCAATAAGCGGAGGAAAAG-3′] and NL4 [forward, 5′-GGTCCGTGTTTCAAGACGG-3′]) PCR analysis and then high-throughput sequencing.

### Library construction and next-generation sequencing (NGS). (i) 16S rRNA gene sequencing.

Two grams of soil was frozen at −80°C prior to DNA extraction with three replicates. Total genome DNA from the soil sample was extracted using the Magen Hipure soil DNA kit (lot no. HE280200) according to the manufacturer’s protocols. DNA was quantified using the Qubit 3.0 fluorometer (Invitrogen, Carlsbad, CA, USA). For each sample, 20 to 30 ng DNA was used to generate amplicons using a MetaVx library preparation kit (Genewiz, South Plainfield, NJ, USA). V3 and V4 hypervariable regions of prokaryotic 16S rRNA genes were selected for generating amplicons and subsequent taxonomy analysis. The V3 and V4 regions were amplified using forward primers containing the sequence CCTACGGRRBGCASCAGKVRVGAAT and reverse primers containing the sequence GGACTACNVGGGTWTCTAATCC. At the same time, indexed adapters were added to the ends of the 16S rRNA gene amplicons to generate indexed libraries ready for downstream NGS on Illumina MiSeq. PCRs were performed in a triplicate 25-μl mixture containing 2.5 μl of TransStart buffer, 2 μl of deoxynucleoside triphosphates (dNTPs), 1 μl of each primer, and 20 ng of template DNA. DNA library concentrations were validated by a Qubit 3.0 fluorometer. Sequencing was performed using a PE250 paired end on an Illumina MiSeq instrument (Illumina, San Diego, CA, USA) by Genewiz.

### (ii) One-cell genome sequencing.

The target MDA products were treated with S1 nuclease (Thermo Fisher Scientific, Waltham, MA, USA) to degrade the single-stranded nucleic acids and then purified by Agencourt AMPure XP beads (Beckman Coulter, Brea, CA, USA). Next-generation sequencing library preparations were constructed following the manufacturer’s protocol (NEBNext Ultra DNA library prep kit for Illumina). For each sample, 1 μg genomic DNA was randomly fragmented to <500 bp by sonication (Covaris S220). The fragments were treated with End Prep enzyme mix for end repairing, 5′ phosphorylation, and dA-tailing in one reaction, followed by a T-A ligation to add adapters to both ends. Size selection of adapter-ligated DNA was then performed using AxyPrep Mag PCR cleanup (Axygen), and fragments of ∼410 bp (with the approximate insert size of 350 bp) were recovered. Each sample was then amplified by PCR for 8 cycles using P5 and P7 primers, with both primers carrying sequences which can anneal with a flow cell to perform bridge PCR and the P7 primer carrying a six-base index allowing for multiplexing. The PCR products were cleaned up using AxyPrep Mag PCR cleanup (Axygen), validated using an Agilent 2100 Bioanalyzer (Agilent Technologies, Palo Alto, CA, USA), and quantified by a Qubit 2.0 fluorometer (Invitrogen, Carlsbad, CA, USA).

Then, libraries with different indexes were multiplexed and loaded on an Illumina HiSeq instrument (Illumina, San Diego, CA, USA). Sequencing was carried out using a 2 × 150 paired-end (PE) configuration; image analysis and base calling were conducted by the HiSeq control software (HCS)+OLB+GAPipeline-1.6 (Illumina). Samples were quantified using a Qubit 2.0 fluorometer (Invitrogen, Carlsbad, CA, USA).

### Sequencing data analysis. (i) 16S rRNA gene sequencing.

The software package QIIME (version 1.9.1) (65) was used for 16S rRNA gene data analysis. The forward and reverse read pairs with minimum overlapping bases of 20 bp were joined and assigned to samples based on barcode and truncated by cutting off the barcode, primer sequence, and low-quality bases (5′ end; quality score <20). Quality filtering on joined sequences was performed, and sequences which did not fulfill the following criteria were discarded: sequence length >200 bp, no ambiguous bases. Then, the chimeric sequences were removed using the UCHIME algorithm (66). Putative contaminants were removed from data sets, as were singletons. Subsequently, the remaining high-quality reads were grouped into operational taxonomic units (OTUs) using the Vsearch algorithm (67) and aligned using default parameters against the Silva_132 database (68). Representative sequences for the shared OTUs, as defined by 97% similarity, were obtained. Relative abundances of the bacterial taxa at the phylum, class, order, family, genus, and species levels were calculated and compared, respectively.

### (ii) One-cell genome analysis.

A dedicated computational pipeline for the single-microbial-cell genome was developed (https://github.com/gongyh/nf-core-scgs) to efficiently analyze single-cell amplified bacterial genome (SAG) data sets by integrating various tools with Nextflow (69). Briefly, reads that passed Illumina’s chastity filter were first quality checked using FastQC (https://www.bioinformatics.babraham.ac.uk/projects/fastqc/) and then quality trimmed using Trim Galore (https://www.bioinformatics.babraham.ac.uk/projects/trim_galore/) in paired end mode for each sample. To detect contaminated DNA fragments, clean reads were phylogenetically classified using Kraken (70). Clean reads were then assembled into contigs using SPAdes (71) in single-cell mode. Taxonomic composition of assembled contigs (longer than 200 bp) was visualized using BlobTools (72). Assembled genomes were annotated using Prokka (73), KofamKOALA (74), and eggNOG-mapper (75). Considering the possibility of DNA contamination for environmental samples, assembled contigs were further split into bins by taxonomic annotations (in the genus level) for each SAG, followed by estimation of genome completeness using CheckM (38). Since a fraction of contigs (especially fragments from plasmids) cannot be assigned to proper taxa, the whole SAG assembly was used to recover metabolic pathways. For the SAG of CRG1, Quast v5.0.2 (76) was used to align contigs to the reference plasmid (with parameters “-m 200 –min-identity 80”), and the alignments were visualized by pyGenomeTracks (77).

To trace the originated species of the SAGs derived from the mock community, reads were also screened using FastQ Screen (78) against a customized database which includes the genomic sequences of E. coli K-12 (NC_000913.3), H. pylori ATCC 26695 (NC_000915.1), *S. elongatus* PCC7942 (NC_007604.1 and NC_007595.1), S. cerevisiae BY4742 (GCF_000146045.2), PhiX (NC_001422.1), Lambda, vector (the UniVec database), and adapters.

### Data availability.

The sequence data reported in this study have been deposited to the NCBI SRA database (PRJNA646329, PRJNA640996, PRJNA640983, and PRJNA669567).
