# Supplementary figures and images for: One-Cell Metabolic Phenotyping and Sequencing of Soil Microbiome by Raman-Activated Gravity-Driven Encapsulation (RAGE)
Source: mSystems. 2021 May 27;6(3):e00181-21. doi: 10.1128/mSystems.00181-21 (PMC8269212; doi:10.1128/mSystems.00181-21)

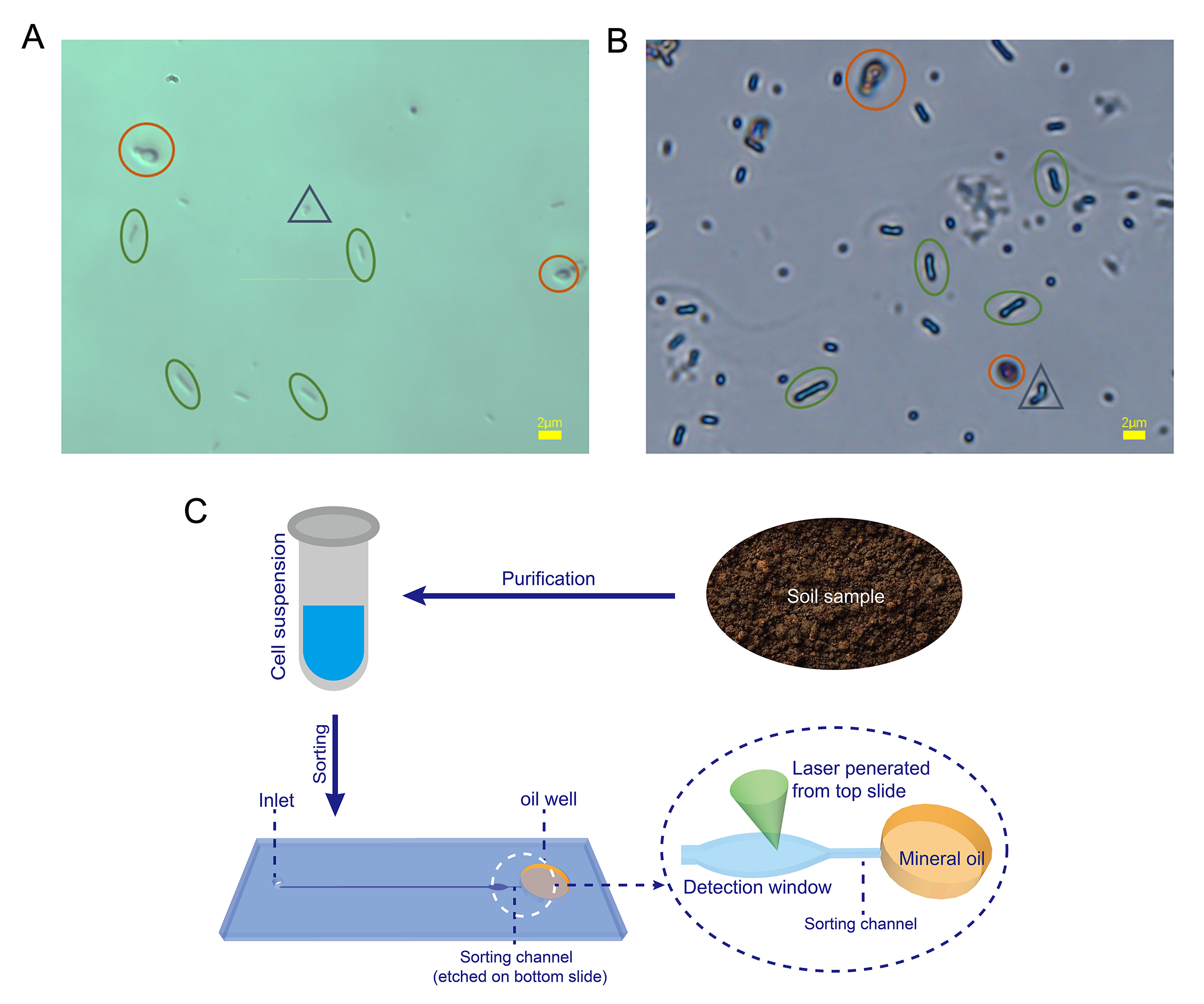

Supplement: FIG S1 [file msystems.00181-21-sf001.tif]

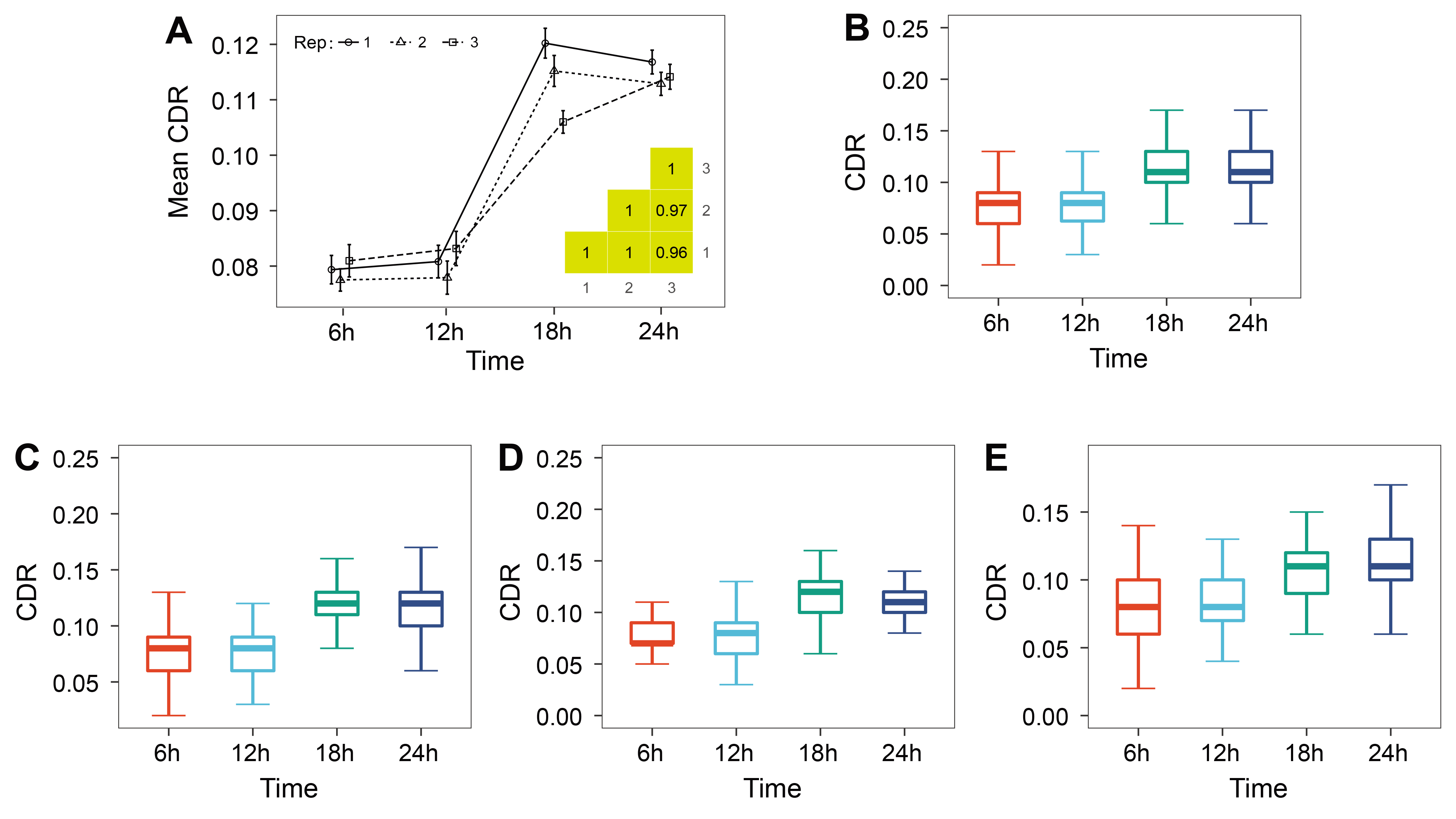

Supplement: FIG S2 [file msystems.00181-21-sf002.tif]

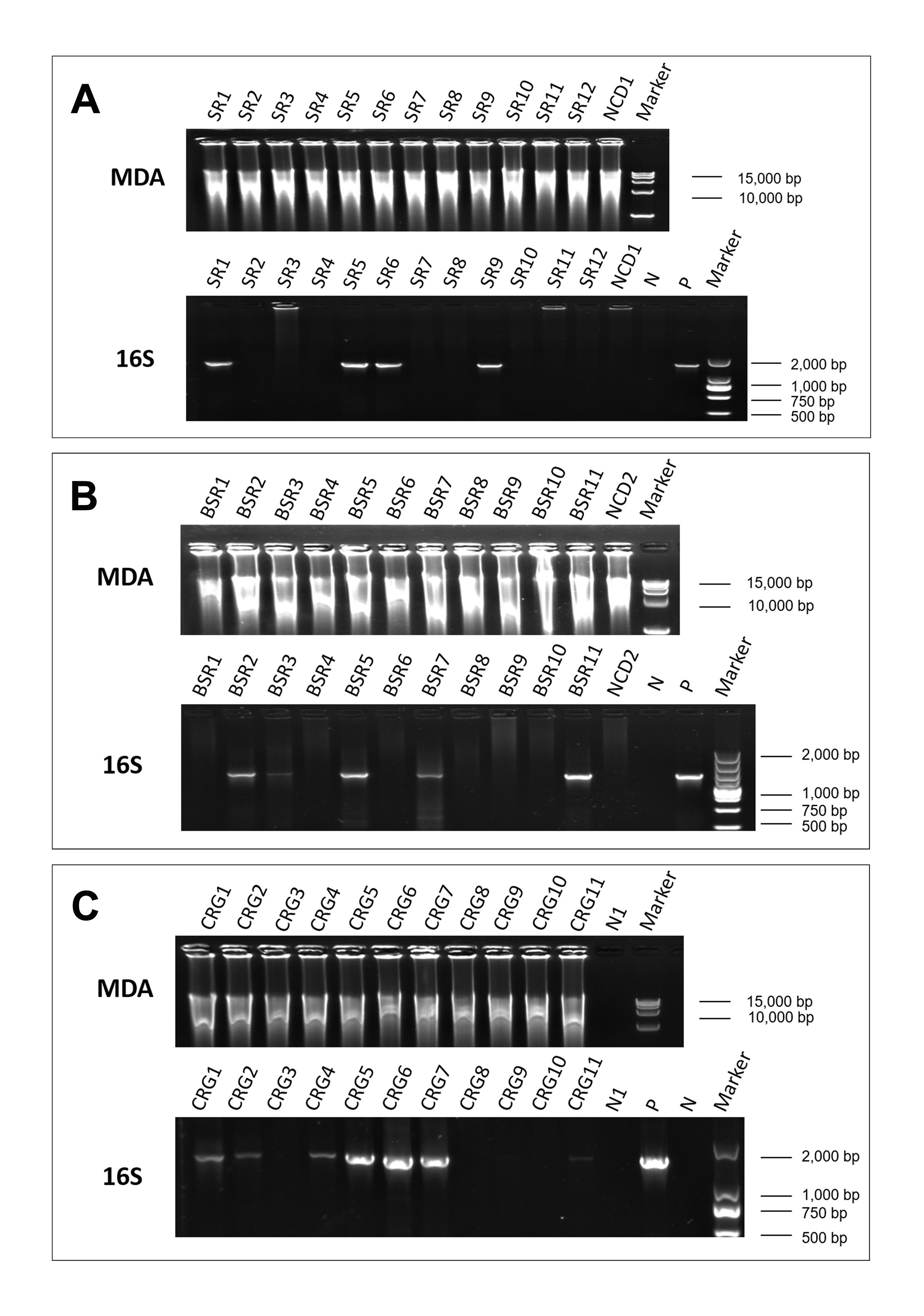

Supplement: FIG S3 [file msystems.00181-21-sf003.tif]

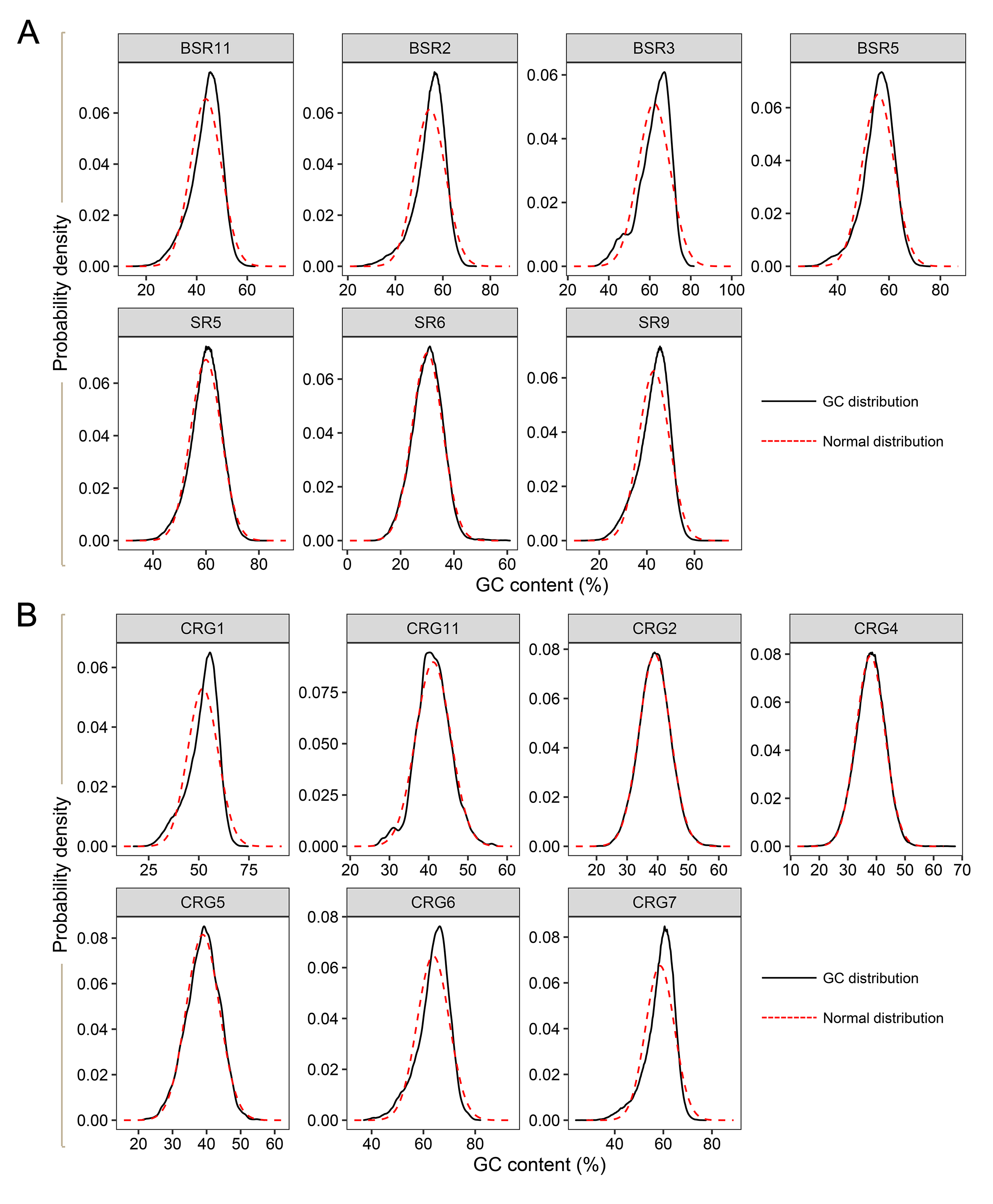

Supplement: FIG S4 [file msystems.00181-21-sf004.tif]

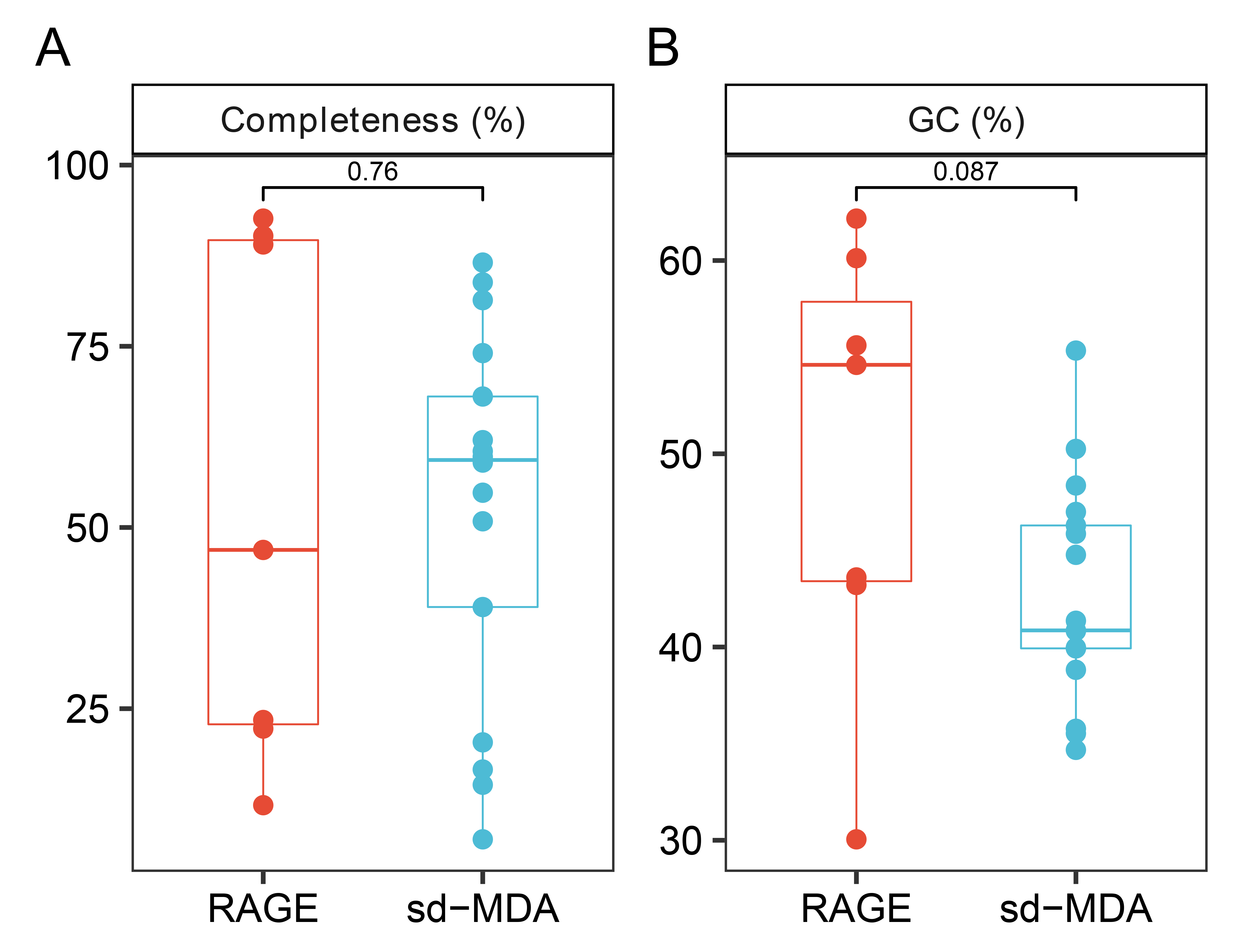

Supplement: FIG S5 [file msystems.00181-21-sf005.tif]
