# Supplementary material for: One-Cell Metabolic Phenotyping and Sequencing of Soil Microbiome by Raman-Activated Gravity-Driven Encapsulation (RAGE)
Source: mSystems. 2021 May 27;6(3):e00181-21. doi: 10.1128/mSystems.00181-21 (PMC8269212; doi:10.1128/mSystems.00181-21)
Supplement: TABLE S1 [file msystems.00181-21-st001.docx]

**Table S1. Sequencing and assembly statistics for single-cell genomes produced by RAGE-Seq.**

| **RAGE-**  **sorted samples** | | **Sequencing** | | **Assembly** | | |
| --- | --- | --- | --- | --- | --- | --- |
|  |  | **Raw reads**  **(Million)** | **Clean reads**  **(Million)** | **Assembly size (Mbp)** | **Number of contigs** | **N50** |
| **CD**  **peak-containing cells** | **SR5** | 11.16*2 | 11.16*2 | 7.75 | 11400 | 1423 |
|  | **SR6** | 11.79*2 | 11.78*2 | 5.03 | 12892 | 279 |
|  | **SR9** | 12.32*2 | 11.31*2 | 9.46 | 18837 | 560 |
|  | **BSR2** | 11.81*2 | 11.79*2 | 5.50 | 4168 | 4846 |
|  | **BSR3** | 12.04*2 | 12.02*2 | 1.88 | 2931 | 996 |
|  | **BSR5** | 13.26*2 | 13.23*2 | 3.19 | 3525 | 2096 |
|  | **BSR11** | 11.87*2 | 11.84*2 | 3.15 | 1957 | 10330 |
| **Carotenoid-producing**  **cells** | **CRG1** | 9.05*2 | 9.03*2 | 58.61 | 62312 | 2715 |
|  | **CRG2** | 8.91*2 | 8.89*2 | 28.25 | 27507 | 2937 |
|  | **CRG4** | 8.80*2 | 8.79*2 | 19.01 | 17504 | 3475 |
|  | **CRG5** | 9.01*2 | 8.99*2 | 2.01 | 1914 | 5491 |
|  | **CRG6** | 13.00*2 | 12.97*2 | 1.80 | 3590 | 631 |
|  | **CRG7** | 9.71*2 | 9.69*2 | 3.16 | 3758 | 2038 |
|  | **CRG11** | 9.05*2 | 9.03*2 | 2.80 | 3141 | 2591 |
