# Supplementary material for: One-Cell Metabolic Phenotyping and Sequencing of Soil Microbiome by Raman-Activated Gravity-Driven Encapsulation (RAGE)
Source: mSystems. 2021 May 27;6(3):e00181-21. doi: 10.1128/mSystems.00181-21 (PMC8269212; doi:10.1128/mSystems.00181-21)
Supplement: TABLE S2 [file msystems.00181-21-st002.docx]

**Table S2.** **Comparison of instruments, techniques and their performance for Raman-activated cell sorting and genome (RACS-Seq) among studies.**

| **No.** | **Paper citations** | **Maker of Raman spectrometer (model)** | **Maker of the cell sorting chip** | **Sorting technique** | **Sample** | **Cell number** | **Genome coverage in microbial RACS-Seq** |
| --- | --- | --- | --- | --- | --- | --- | --- |
| 1 | Raman activated cell ejection for isolation of single cells, *Anal Chem*, 2013. (1) **(Ref. 30 in former version)** | Horiba (LabRAM HR) | This team in collaboration with Wei E. Huang’s Lab at Univ. of Sheffield | RACE | Pure culture of *E. coli* DH5α | 20 bacterial cells per tube | No post-sorting genome sequencing was demonstrated |
| 2 | Single-cell genomics based on Raman sorting reveals novel carotenoids-containing bacteria in the Red sea, *Microb Biotechnol*, 2017. (2) | Horiba (LabRAM HR; customized) | Wei E. Huang’s Lab at Univ. of Oxford | RACE | Marine microbiome | 1~8 bacterial cells per tube | Three post-RACE 1-cell sequencing reactions were at 8.18%, 6.65%, 4.17% respectively, and for 8-cell reactions the coverages were 8.95% and 19.29% respectively |
| 3 | Raman-activated cell sorting and metagenomics sequencing reveals carbon-fixing bacteria in the ocean, *Environ Microbiol*, 2018. (3) | Horiba (LabRAM HR; customized) | This team | RACE | Marine microbiome | 30 bacterial cells per tube | Maximally 13.66% |
| 4 | Rational Optimization of Raman-activated Cell Ejection and Sequencing for Bacteria, *Anal Chem*, 2020. (4) **(Ref. 29 in former version)** | Horiba (LabRAM HR; customized) | This team (in collaboration with QSB*) | RACE | Pure culture of lab-model *E. coli*, and soil microbiome | 2~5 bacterial cells per tube | For lab-model *E. coli* pure culture: maximally 94.78% (5 cells per tube)  For soil microbiome: maximally 26.42% for 2~5 cells per tube (all 1-cell-per-tube samples failed in sequencing) |
| 5 | Phenome-genome profiling of single bacterial cell by Raman-activated gravity-driven encapsulation and sequencing, *Small*, 2020. (5) **(Ref. 33 in former version)** | QSB* (RACS-Seq) | This team (in collaboration with QSB*) | RAGE | Pure culture *E. coli* ATCC 35218; clinical *E. coli* cells directly from patient urine sample | Precisely one bacterial cell per tube | Maximally 99.50% |
| 6 | This work | QSB* (RACS-Seq) | This team (in collaboration with QSB*) | RAGE | Pure culture of mock community (*E. coli* K-12 DH5α, *H. pylori* ATCC26695, *S. elongatus* PCC7942 and *S. cerevisiae* BY4742) and soil microbiome | Precisely one bacterial cell per tube | For lab-model mock community: maximally 99.73% (one-cell-per-tube)  For soil microbiome: maximally 92.62% (one-cell-per-tube) |
| 7 | Positive dielectrophoresis based Raman-activated droplet sorting for culture-free and label-free screening of enzyme function in vivo, *Sci Adv*, 2020. (6) | QSB* (FlowRACS) | This team (in collaboration with QSB*) | pDEP-RADS | Pure culture *S. cerevisiae* strain H1246 | Not applicable | No post-sorting genome sequencing was demonstrated |

*: Qingdao Single-cell Biotech Inc., Qingdao, Shandong, China (<http://www.singlecellbiotech.com>).

**References**

1. Wang Y, Ji Y, Wharfe ES, Meadows RS, March P, Goodacre R, Xu J, Huang WE. 2013. Raman activated cell ejection for isolation of single cells. Anal Chem 85:10697-10701.

2. Song Y, Kaster AK, Vollmers J, Song Y, Davison PA, Frentrup M, Preston GM, Thompson IP, Murrell JC, Yin H. 2017. Single‐cell genomics based on Raman sorting reveals novel carotenoid‐containing bacteria in the Red Sea. Microb Biotechnol 10:125-137.

3. Jing X, Gou H, Gong Y, Su X, Xu L, Ji Y, Song Y, Thompson IP, Xu J, Huang WE. 2018. Raman-activated cell sorting and metagenomic sequencing revealing carbon-fixing bacteria in the ocean. Environ Microbiol 20:2241-2255.

4. Su X, Gong Y, Gou H, Jing X, Xu T, Zheng X, Chen R, Li Y, Ji Y, Ma B, Xu J. 2020. Rational optimization of Raman-activated cell ejection and sequencing for bacteria. Anal Chem 12:8081-8089.

5. Xu T, Gong Y, Su X, Zhu P, Dai J, Xu J, Ma B. 2020. Phenome-genome profiling of single bacterial cell by Raman-activated gravity-driven encapsulation and sequencing. Small:e2001172.

6. Wang X, Xin Y, Ren L, Sun Z, Zhu P, Ji Y, Li C, Xu J, Ma B. 2020. Positive dielectrophoresis–based Raman-activated droplet sorting for culture-free and label-free screening of enzyme function in vivo. Sci Adv 6:eabb3521.

7. Hosokawa M, Nishikawa Y, Kogawa M, Takeyama H. 2017. Massively parallel whole genome amplification for single-cell sequencing using droplet microfluidics. Sci Rep 7:1-11.
